# Identification of Prohibitin (PHB) as an Entry Factor of Highly Pathogenic Coronaviruses Using Metabolic Glycoengineering and Photo-Crosslinking Techniques

**DOI:** 10.1080/22221751.2026.2686461

**Published:** 2026-06-08

**Authors:** Xingxing Zhu, Xiaoyang Wang, Yiming Wang, Xinchen Wang, Yuanhao Li, Liwen Hua, Honggao Duan, Rongding Yan, Tao Yuan, Jing Li, Yeshuang Yuan, Bo Zhang, Demin Zhou, Sulong Xiao

**Affiliations:** a State Key Laboratory of Natural and Biomimetic Drugs, School of Pharmaceutical Sciences, Peking University, Beijing, People’s Republic of China; b Jiangxi Provincial Key Laboratory of Prevention and Treatment of Infectious Diseases, Jiangxi Medical Center for Critical Public Health Events, The First Affiliated Hospital, Jiangxi Medical College, Nanchang University, Nanchang, People’s Republic of China; c China Southwest United Graduate School, Kunming, People’s Republic of China; d Yunnan Baiyao International Medical Research Center, Peking University, Beijing, People’s Republic of China; e Ningbo Institute of Marine Medicine, Peking University, Ningbo, People’s Republic of China; f State Key Laboratory of Complex Severe and Rare Diseases, Peking Union Medical College Hospital, Chinese Academy of Medical Sciences and Peking Union Medical College, Beijing, People’s Republic of China; g National Center for Clinical Laboratories, Institute of Geriatric Medicine, Chinese Academy of Medical Sciences, Beijing Hospital/ National Center of Gerontology, Beijing, People’s Republic of China

**Keywords:** SARS-CoV-2, coronavirus, prohibitin (PHB), photo-crosslinking probe, Ac_4_ManNAz

## Abstract

Coronaviruses (CoVs) rely on host surface factors to mediate viral attachment and entry, yet conserved host cofactors shared across different CoVs remain poorly defined. Here, we established a host-factor capture platform based on metabolic glycoengineering and photo-crosslinking to identify host factors associated with CoV Spike proteins under near-physiological conditions. Using this approach, we identified prohibitin (PHB) as a candidate Spike-associated host factor shared by SARS-CoV, MERS-CoV, and SARS-CoV-2. Functional analyses showed that depletion of membrane-associated PHB decreased pseudovirus infection, whereas PHB reconstitution or overexpression enhanced susceptibility across multiple cell systems, including Huh7, BEAS-2B, Calu-3, and primary human airway epithelial cells (hAECs). In addition, antibody-mediated blockade of cell-surface PHB suppressed pseudovirus entry in both hAECs and human lung organoids. Immunohistochemical analyses further demonstrated PHB expression in normal human lung tissues. Mechanistically, recombinant PHB interacted with the Spike proteins of all three CoVs, and this association was supported by surface plasmon resonance and co-immunoprecipitation assays. Structural prediction combined with fragment-based alanine scanning identified residues 139–154 within the extracellular PHB domain as a critical region for efficient Spike association. Furthermore, PHB-associated pseudovirus entry was sensitive to lipid raft disruption, supporting a role for cholesterol-dependent membrane microdomains in this process. Together, these findings identify membrane-associated PHB as a conserved Spike-associated host factor linked to CoV entry.

## GRAPHICAL ABSTRACT

**Figure:**
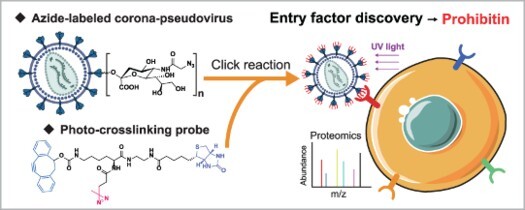

## Introduction

Coronaviruses (CoVs), including severe acute respiratory syndrome coronavirus (SARS-CoV), Middle East respiratory syndrome coronavirus (MERS-CoV), and severe acute respiratory syndrome coronavirus 2 (SARS-CoV-2), have caused multiple outbreaks of severe respiratory diseases in humans [1]. Viral entry is initiated by the coronavirus Spike glycoprotein through receptor engagement and membrane fusion. Although canonical receptors such as angiotensin-converting enzyme 2 (ACE2) for SARS-CoV and SARS-CoV-2 [2–6] and dipeptidyl peptidase 4 (DPP4) for MERS-CoV [7,8] are well established, accumulating evidence indicates that coronavirus entry is coordinated by a broader network of host-associated factors beyond canonical receptor recognition. Multiple auxiliary entry-associated molecules, including transmembrane protease serine 2 (TMPRSS2) [9], heparan sulfate [10], neuropilin-1 (NRP1) [11], cluster of differentiation 147 (CD147) [12], scavenger receptor class B type 1 (SR-B1) [13], metabotropic glutamate receptor 2 (mGluR2) [14], asialoglycoprotein receptor 1 (ASGR1) [15], and tyrosine-protein kinase receptor UFO (AXL) [16], have been implicated in facilitating viral attachment, membrane fusion, or entry efficiency. Nevertheless, whether conserved membrane-associated host factors collectively facilitate the entry of distinct coronaviruses remains incompletely understood.

To identify virus-associated host factors, multiple target discovery approaches have been developed, including comparative genomics [15], CRISPR-based genetic screening [17], and affinity enrichment strategies using chemical probes [18]. Among these, photo-affinity labelling has emerged as a powerful approach for capturing transient virus–host interactions under near-native conditions. Current strategies typically rely on unnatural amino acid incorporation [19] or thiol-reactive chemical modification using reagents such as maleimide [18]. Although these approaches enable photo-crosslinker installation, they may perturb protein structure, alter viral glycoprotein conformation, or introduce nonspecific background interactions, thereby limiting the specificity and robustness of host factor identification. These limitations highlight the need for more biocompatible and minimally perturbative strategies for profiling coronavirus-associated host factors.

1,3,4,6-Tetra-*O*-acetyl-*N*-azidoacetylmannosamine (Ac_4_ManNAz) is an azide-modified metabolic precursor that can be incorporated into glycoprotein glycans through endogenous glycosylation pathways [20,21]. Coronavirus Spike proteins are extensively glycosylated and contain multiple N-linked glycosylation and potential sialylation sites [22], providing a biochemical basis for metabolic glycoengineering-based probe installation. We therefore hypothesized that the dense glycan architecture of coronavirus Spike proteins could be exploited for minimally perturbative installation of photo-crosslinking probes through metabolic glycoengineering and bioorthogonal chemistry, thereby enabling capture of Spike-associated host factors during viral attachment.

**Scheme 1.**
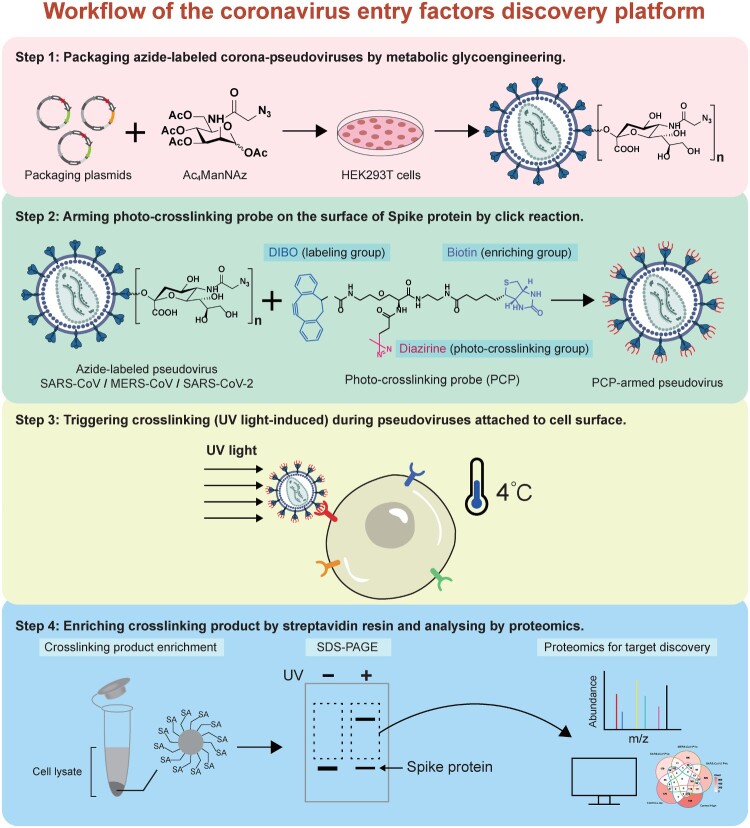
Identification of coronavirus entry-associated host factors using metabolic glycoengineering and photo-crosslinking techniques.

In this study, we established a coronavirus host factor capture platform by integrating metabolic glycoengineering, photo-crosslinking, and proteomic profiling (Scheme 1). Using this strategy, we identified prohibitin (PHB) as a conserved Spike-associated host factor linked to the entry of SARS-CoV, MERS-CoV, and SARS-CoV-2. Functional analyses in transformed cell lines, primary human airway epithelial cells, and human lung organoids further supported the physiological relevance of the PHB-associated entry phenotype. Together, this work establishes a chemically enabled framework for profiling coronavirus-associated host factors and highlights membrane-associated PHB as a conserved factor linked to coronavirus entry.

Photo-crosslinking probes were installed onto glycosylated coronavirus Spike proteins through metabolic glycoengineering and bioorthogonal chemistry. Upon viral attachment, UV-induced crosslinking enabled covalent capture of Spike-proximal membrane-associated proteins for enrichment and proteomic identification of candidate host factors.

## Materials and methods

### Plasmids and cells

Spike expression plasmids were constructed using the pcDNA3.1 backbone. The lentiviral packaging plasmids psPAX2 and pMD2.G were obtained from Addgene. The reporter vector used for pseudovirus production was pLVX-fLuc-2A-GFP. The lentiCRISPRv2-PHB knockout construct containing the sgRNA sequence CAGGATGAGCCCAAAGGTGG and the PHB rescue vector pLVX-CMV-huPHB1-mCherry-Neo were generated from corresponding lentiviral backbones. All constructs were verified by DNA sequencing.

HEK293 T, Huh7, BEAS-2B, Calu-3, and A549 cells were cultured in DMEM or Ham’s F-12 K medium supplemented with 10% fetal bovine serum (FBS). HEK293T-ACE2 and HEK293T-DPP4 cells were established by lentiviral transduction. PHB-knockout Huh7, BEAS-2B, and Calu-3 cell lines were generated using PHB-targeting CRISPR constructs. Primary human airway epithelial cells (hAECs; Procell Biotechnology) and human lung organoids (D1Med Medical Technology) were maintained according to the manufacturers’ instructions.

### Compounds and reagents

Ac_4_ManNAz and the photo-crosslinking probe were synthesized as previously reported [23]. Neutralizing antibodies against SARS-CoV, MERS-CoV, and SARS-CoV-2 were obtained from Sino Biological. Antibodies against PHB (Abcam, Cat# ab75771), PHB2 (Cat# ab182139), ACE2 (Cat# ab272500), DPP4 (Cat# ab222716), TMPRSS2 (Cat# ab109131), and NRP1 (Cat# ab81321) were purchased from Abcam. Antibodies against coronavirus Spike proteins and biotin-HRP were obtained from Cell Signaling Technology. Recombinant stabilized trimeric Spike proteins, ACE2, and DPP4 were obtained from ACRO Biosystems, whereas recombinant PHB (Cat# ab140551) and PHB2 (Cat# ab167836) proteins were purchased from Abcam. AF488-NHS and Matrigel were obtained from Aladdin and Corning, respectively.

### Packaging of pseudoviruses and probe arming

Coronavirus pseudoviruses were generated in HEK293 T cells by co-transfection of Spike expression plasmids, psPAX2 packaging plasmids, and pLVX-fLuc-2A-GFP reporter vectors using MegaTran 2.0 (OriGene Technologies). Ac_4_ManNAz (100 μM) was added 6 h post-transfection for metabolic labelling. Culture supernatants were collected at 48–72 h, clarified by centrifugation and filtration, concentrated, and stored at −80 °C. Bald pseudoviruses were generated in parallel without Spike plasmids. For probe arming, concentrated pseudoviruses were incubated overnight at 4 °C with photo-crosslinking probes (100 μM final concentration) in the dark, followed by ultrafiltration to remove excess probes. For transmission electron microscopy (TEM), naked pseudoviruses were negatively stained with phosphotungstic acid and imaged using a JEM-1400PLUS microscope (JEOL Ltd.).

### Neutralization assay and determination of neutralization curves

Neutralizing activity was assessed using a pseudovirus-based microneutralization assay. HEK293T-ACE2 or HEK293T-DPP4 cells were seeded in 96-well plates and infected with SARS-CoV, MERS-CoV, or SARS-CoV-2 Spike pseudoviruses in the presence of serially diluted neutralizing antibodies. After 72 h of incubation, luciferase activity was measured using the Steady-Lumi^TM^ II Firefly Luciferase Assay Kit (Beyotime) as an indicator of viral infection. Each condition was tested in triplicate, and neutralization curves were generated based on relative luminescence units (RLU).

### Photo-crosslinking and mass spectrometry analysis

Photo-crosslinking probe–armed or naked pseudoviruses were concentrated by ultracentrifugation and normalized based on p24 antigen levels prior to infection. Huh7 cells were incubated with pseudoviruses (MOI = 0.2) at 4 °C for 2 h to permit viral attachment while preventing endocytosis, followed by extensive phosphate-buffered saline (PBS) washing to remove unbound particles. Cells were subsequently maintained either under dark conditions or exposed to 365-nm UV irradiation on ice for 15 min to induce photo-crosslinking.

Following UV treatment, cells were lysed in RIPA buffer supplemented with protease inhibitors, and clarified lysates were incubated overnight with streptavidin affinity resin at 4 °C. Resin-bound proteins were eluted by boiling, separated by SDS-PAGE, and visualized by Coomassie brilliant blue staining. Protein bands above the molecular weight of Spike were excised and subjected to label-free LC–MS/MS analysis. Protein identification and relative quantification were performed against the UniProt human protein database using a label-free quantification (LFQ) workflow. Proteins reproducibly enriched in UV-treated samples relative to non-UV controls were considered candidate Spike-associated host factors. All experiments were independently repeated at least three times. Proteins with a UV-to-non-UV fold change >1.5 and a false discovery rate (FDR) ≤ 20% were considered significantly enriched.

### Expression analysis of coronavirus receptors and PHB in different cell lines

PHB localization in Huh7 cells was analyzed by immunofluorescence staining using anti-PHB antibody (Abcam, Cat# ab75771) and AF488-conjugated secondary antibody. Nuclei and plasma membranes were counterstained with Hoechst and DiD, respectively, and images were acquired using a Zeiss LSM 880 confocal microscope. Flow cytometric analysis was performed using a CytoFLEX S flow cytometer (Beckman Coulter). Total cellular expression levels of ACE2, DPP4, TMPRSS2, NRP1, and PHB were analyzed by western blotting using whole-cell lysates prepared in RIPA buffer supplemented with protease inhibitors. GAPDH served as the loading control. For membrane-associated protein analysis, cell surface proteins were labelled with Sulfo-NHS-SS-Biotin (Thermo Fisher Scientific, Cat# 21445), enriched using streptavidin magnetic beads, and analyzed by western blotting. Na^+^/K^+^-ATPase was used as the membrane protein loading control.

### PHB knockdown, knockout, and rescue

PHB knockdown was achieved by siRNA transfection using Lipofectamine RNAiMAX (Thermo Fisher Scientific). PHB knockout cell lines were generated by lentiviral CRISPR/Cas9 transduction followed by puromycin selection. Lentiviral particles were produced by co-transfecting HEK293T cells with the lentiCRISPRv2-PHB construct, psPAX2, and pMD2.G plasmids. For rescue experiments, PHB-knockout cells were transduced with sgRNA-resistant codon-modified PHB lentiviral constructs and selected with neomycin to establish stable rescue cell lines.

### Surface plasmon resonance (SPR) experiments

Binding affinities between coronavirus Spike proteins and PHB were measured using a Biacore 8 K SPR system (Cytiva). PHB, ACE2, DPP4, or PHB2 was immobilized on a CM5 sensor chip via standard amine coupling using PBS-P running buffer. Serial dilutions of Spike proteins were injected, and equilibrium dissociation constants (K_D_) were calculated using a 1:1 Langmuir binding model in Biacore Insight Evaluation Software (version 5.0.18).

### Primary human airway epithelial cell (hAEC) culture and genetic manipulation

Primary human airway epithelial cells (hAECs; Cat# CP-H209, Procell Biotechnology Ltd.) were cultured in human alveolar epithelial cell complete medium (Cat# CM-H209) at 37 °C with 5% CO_2_. PHB knockdown (KD) was achieved by siRNA transfection using Lipofectamine RNAiMAX (Thermo Fisher Scientific), whereas PHB overexpression (OE) was established using lentiviral transduction with pLVX-CMV-huPHB1-mCherry-Neo particles. Parental, PHB-KD, and PHB-OE hAECs were infected with HIV-1–based pseudoviruses bearing SARS-CoV, MERS-CoV, or SARS-CoV-2 Spike proteins. Viral entry efficiency was quantified 24 h post-infection by firefly luciferase assay (Beyotime).

### Dose-dependent antibody inhibition assay

Huh7 cells or hAECs were seeded in 96-well plates and preincubated for 1 h with serially diluted antibodies against PHB, ACE2, DPP4, or PHB2 (1,000–0.001 nM). Cells were subsequently infected with coronavirus pseudoviruses in the continued presence of antibodies for 24 h, followed by luciferase-based infection quantification. Cell viability was evaluated in parallel using the CellTiter-Glo assay (Promega) according to the manufacturer’s instructions.

### Human lung organoid culture and antibody blocking assays

Human lung organoids (D1Med Medical Technology Ltd.) were maintained in lung organoid culture medium (Cat# K212M10) according to the manufacturer’s instructions. For pseudovirus attachment assays, coronavirus pseudoviruses were labelled with Alexa Fluor 488 NHS ester (AF488-NHS; Aladdin) and purified by ultrafiltration to remove excess fluorophore.

Lung organoids were dissociated from Matrigel using trypsin-EDTA and incubated with anti-PHB (Abcam, Cat# ab75771), anti-ACE2 (ab272500), anti-DPP4 (ab222716), or anti-PHB2 (ab182139) antibodies (10 nM) for 1 h at 37 °C prior to incubation with AF488-labelled pseudoviruses. After washing, viral attachment was visualized by confocal microscopy following Hoechst 33342 nuclear staining.

For flow cytometric quantification, organoids were further dissociated into single-cell suspensions, filtered through a 70 μm strainer, and analyzed using a CytoFLEX S flow cytometer (Beckman Coulter). Mean fluorescence intensity (MFI) of AF488-positive signals was quantified.

### Co-immunoprecipitation of PHB and coronavirus Spike proteins

HEK293 T cells were transfected with HA-tagged PHB (wild type or mutants) overexpression plasmids as described above. After 48 h, cells were infected with pseudovirus (MOI = 1) for 24 h at 37 °C. Cells were washed with PBS, lysed on ice with IP lysis buffer, and centrifuged at 14,000 × g for 15 min to obtain supernatants. Supernatants were incubated with anti-HA tag antibody or mouse IgG agarose overnight at 4 °C. Agarose beads were washed three times with IP lysis buffer, eluted, and analyzed by western blotting.

### Immunohistochemical analysis of human lung tissues

Formalin-fixed paraffin-embedded (FFPE) lung tissue sections from eight independent donors were subjected to immunohistochemical (IHC) staining using anti-PHB antibody (Abcam, Cat# ab75771; 1:500), followed by HRP-conjugated secondary antibody incubation, DAB visualization, and hematoxylin counterstaining. Images were acquired using a bright-field microscope imaging system. Human lung tissue collection and related analyses were approved by the Medical Research Ethics Committee of the First Affiliated Hospital of Nanchang University [approval number: (2025)CDYFYYLK(12-017)]. Written informed consent was obtained from all donors or their legal representatives.

### Public transcriptomic analysis of human tissue expression

Public transcriptomic datasets were retrieved from the Expression Atlas database (experiment E-MTAB-2836), which contains RNA-seq profiles from 122 human donors across 32 tissue types. TPM-normalized expression values for PHB, PHB2, ACE2, DPP4, TMPRSS2, and NRP1 were extracted for comparative tissue distribution analysis. Averaged TPM values across tissue types were used to generate heatmaps and tissue expression plots illustrating the relative abundance of the indicated genes in different human tissues.

### Structure prediction of Spike–PHB complexes using AlphaFold 3

The amino acid sequences of the Spike glycoproteins from SARS-CoV-2, SARS-CoV, and MERS-CoV, together with human PHB1 and PHB2, were retrieved from the UniProt database. Structural modelling was performed using AlphaFold 3 multimer in a two-step strategy. Because the native PHB complex is a large hetero-11-mer assembly composed of six PHB1 subunits and five PHB2 subunits, direct prediction of the full complex with trimeric Spike proteins was not computationally feasible. Therefore, in the first step, the trimeric Spike ectodomain of each coronavirus was modelled in complex with PHB1 to identify the putative PHB-binding region. These preliminary predictions consistently localized the interaction to the RBDs. In the second step, the isolated RBD of each Spike protein was modelled in complex with the 6×PHB1/5×PHB2 PHB hetero-11-mer, and the resulting models were used for subsequent interface analysis. Model confidence was evaluated using the predicted local distance difference test (pLDDT), and the final structures were visualized in PyMOL.

### Lipid raft disruption and restoration of BEAS-2B and Huh7

Lipid raft integrity in BEAS-2B and Huh7 cells was disrupted by treatment with methyl-β-cyclodextrin (MβCD), a cholesterol-depleting agent. Briefly, cells were incubated with MβCD at a final concentration of 10 mM in serum-free medium at 37 °C for 30 min to extract membrane cholesterol and disassemble lipid raft microdomains. Cell viability after MβCD treatment was monitored. After treatment, cells were washed three times with PBS and maintained in fresh medium for subsequent experiments.

For lipid raft restoration, cholesterol was replenished by incubating MβCD-treated cells with water-soluble cholesterol (cholesterol–MβCD complex) at a final cholesterol concentration of 100 µg/mL for 60 min at 37 °C. Following cholesterol repletion, cells were washed with PBS to remove excess cholesterol prior to further analysis.

To evaluate lipid raft distribution, cells were incubated with FITC-CTxB at 4 °C for 20 min. After washing with cold PBS, nuclei were counterstained with Hoechst 33342 (1:500 dilution) for 10 min at room temperature. Cells were then washed again with PBS and fluorescence distribution on the plasma membrane was visualized using confocal laser scanning microscopy.

## Results

### Construction and characterization of metabolically labelled coronavirus pseudoviruses

**Figure 1.**
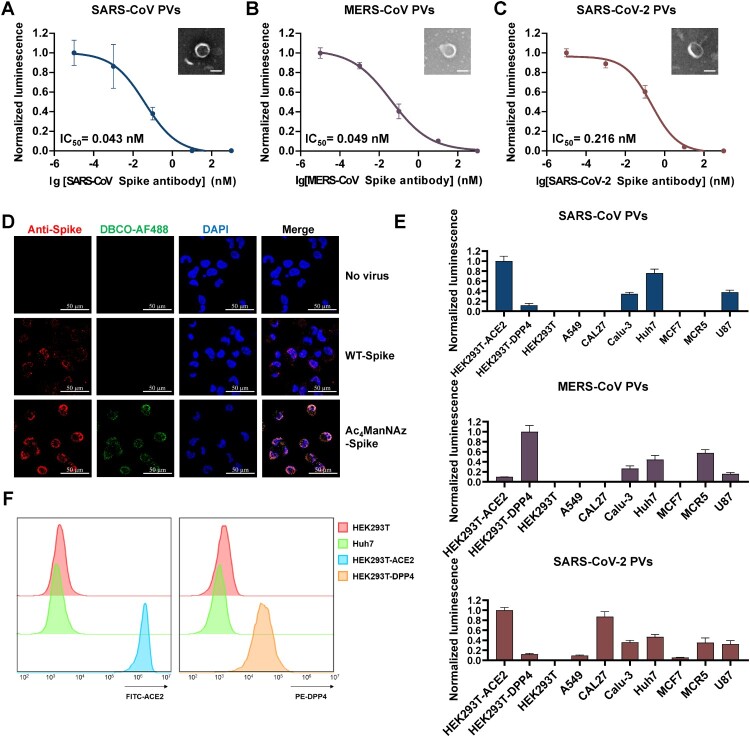
**Metabolic azide labelling of coronavirus pseudoviruses and selection of a target cell model for virus-binding analysis**. (A–C) Neutralization of HIV-1–based pseudoviruses bearing the Spike proteins of SARS-CoV (A), MERS-CoV (B), and SARS-CoV-2 (C) using corresponding Spike-specific antibodies. Dose–response curves were used to validate pseudovirus infectivity and antigenic integrity. Insets show representative transmission electron microscopy (TEM) images of pseudoviruses. Data are presented as mean ± SD (n = 3). Scale bars, 100 nm. (D) Colocalization of Spike proteins and metabolically labelled viral glycans in pseudoviruses produced in the presence of Ac_4_ManNAz. Azide-modified glycoconjugates were conjugated with DBCO-AF488 (green), and Spike proteins were detected by immunofluorescence staining (red). Nuclei were counterstained with DAPI (blue). “No virus” and unlabelled WT-Spike pseudoviruses served as controls. Scale bars, 50 μm. (E) Cell tropism profiling of SARS-CoV, MERS-CoV, and SARS-CoV-2 pseudoviruses across a panel of human-derived cell lines. Relative infection efficiencies were quantified by normalized luminescence signals to identify suitable target cells for subsequent virus-binding experiments. (F) Flow cytometric analysis of ACE2 and DPP4 surface expression in HEK293 T, Huh7, HEK293T-ACE2, and HEK293T-DPP4 cells to support selection of the target cell model for coronavirus pseudovirus binding studies.

To establish a chemically traceable system for studying coronavirus entry, we generated replication-defective HIV-1–based lentiviral pseudoviruses bearing Spike proteins from SARS-CoV, MERS-CoV, or SARS-CoV-2, which enable the investigation of Spike-mediated entry in a safer and less restrictive experimental setting while minimizing confounding effects from post-entry viral replication events. All three pseudoviruses showed strong sensitivity to neutralizing antibodies in validation assays, with IC_50_ values in the low-nanomolar range, confirming the functionality of the incorporated Spike proteins (Figure 1A-C). Transmission electron microscopy further confirmed the expected morphology of the pseudovirus.

To enable selective visualization and capture of viral surface glycans, Ac_4_ManNAz was metabolically incorporated into viral glycoproteins during pseudovirus production, followed by conjugation with a DBCO-linked fluorophore through strain-promoted azide–alkyne cycloaddition (SPAAC). Confocal imaging showed strong colocalization between the fluorescent labelling signal and Spike staining, indicating efficient and selective labelling of Spike-bearing pseudovirus (Figure 1D).

We next screened a panel of human-derived cell lines for susceptibility to SARS-CoV, MERS-CoV, and SARS-CoV-2 pseudovirus. Among them, Huh7 subline was selected because it supported efficient infection by all three pseudoviruses (Figure 1E). Flow cytometric analysis showed that surface ACE2 and DPP4 were barely detectable in this subline (Figure 1F). This cell line was therefore selected for subsequent experiments aimed at identifying additional host factors involved in coronavirus entry.

### Development of a photo-crosslinking platform for coronavirus host factor capture

Building on the efficient metabolic labelling of Spike glycans, we established a photo-crosslinking platform for proximity-dependent capture of virus-associated host proteins (Figure 2A). The multifunctional probe contained a dibenzocyclooctyne (DIBO) group for SPAAC conjugation, a diazirine moiety for UV-induced crosslinking, and a biotin handle for enrichment.

**← Figure 2.**
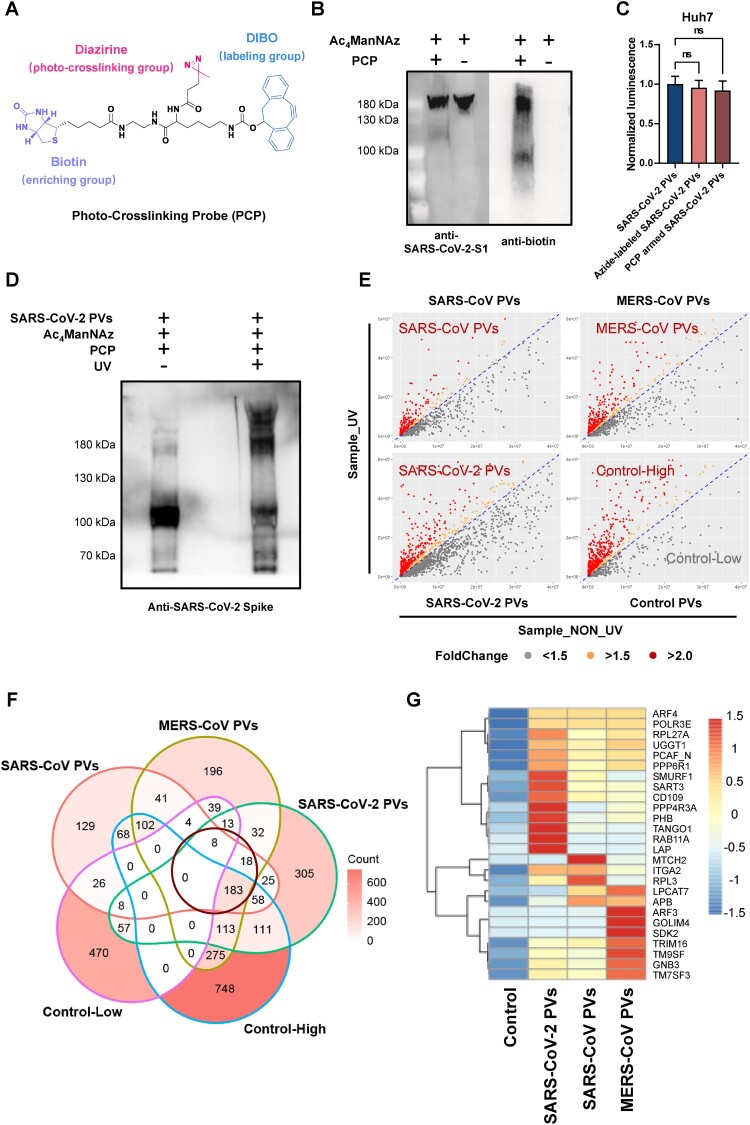
**Photo-crosslinking-assisted capture and proteomic profiling of coronavirus Spike-proximal host factors.** (A) Structure and modular design of the photo-crosslinking probe (PCP), comprising a diazirine group for UV-induced crosslinking, a DIBO moiety for conjugation to azide-labelled viral glycans, and a biotin tag for affinity enrichment. (B) Validation of PCP conjugation to metabolically labelled SARS-CoV-2 pseudoviruses. Immunoblotting with anti-Spike and anti-biotin antibodies confirmed selective biotin labelling of Ac_4_ManNAz-treated pseudoviruses. (C) Effects of Ac_4_ManNAz labelling and PCP conjugation on SARS-CoV-2 pseudovirus infectivity in Huh7 cells. Neither modification significantly affected pseudovirus entry efficiency. Data are presented as mean ± SD; ns, not significant. (D) UV-dependent capture of Spike-associated host protein complexes. PCP-conjugated SARS-CoV-2 pseudoviruses were incubated with target cells followed by UV irradiation. Immunoblotting revealed increased high-molecular-weight Spike-containing species in UV-treated samples, consistent with covalent capture of heterogeneous Spike-proximal membrane-associated complexes. (E) Quantitative proteomic analysis of host proteins enriched by SARS-CoV, MERS-CoV, and SARS-CoV-2 pseudoviruses. Scatter plots compare UV-irradiated and non-irradiated samples, with enriched proteins highlighted based on fold-change thresholds. Envelope-deficient pseudoviruses were included to define background enrichment. (F) Overlap analysis of host proteins enriched by SARS-CoV, MERS-CoV, and SARS-CoV-2 pseudoviruses relative to control groups, identifying shared and virus-preferential candidate host factors. (G) Heatmap of representative host factors enriched in coronavirus pseudovirus capture samples, showing distinct and shared enrichment profiles across SARS-CoV-2, SARS-CoV, MERS-CoV, and control groups.

Efficient probe conjugation to azide-labelled pseudoviruses was confirmed by biotin-specific detection at the expected Spike molecular weight (Figure 2B). Importantly, probe labelling did not significantly affect pseudovirus infectivity (*P* > 0.05, Figure 2C), indicating preservation of Spike-mediated entry activity. Time-course analysis further identified experimental conditions that supported robust virus–cell association with minimal perturbation (Fig. S1).

To evaluate the specificity of this strategy, probe-conjugated SARS-CoV-2 pseudoviruses were incubated with HEK293T-ACE2 cells under crosslinking conditions. UV irradiation resulted in the selective formation of Spike-associated ACE2-containing complexes (Fig. S2), supporting the feasibility and specificity of this approach for capturing Spike-proximal host factors.

### Crosslinking-assisted proteomic identification of PHB as a coronavirus Spike-associated host factor

Photo-crosslinking experiments in Huh7 cells using SARS-CoV-2 pseudoviruses revealed UV-dependent high-molecular-weight Spike-containing species (>180 kDa), consistent with covalent crosslinking between viral Spike proteins and proximal host cell surface-associated components during viral attachment (Figure 2D). In non-irradiated controls, only monomeric Spike (∼100 kDa) was detected. Gel regions above the monomeric Spike band were excised and subjected to mass spectrometric analysis to identify candidate host factors. Parallel Spike-proximal host protein capture experiments were performed using SARS-CoV and MERS-CoV pseudoviruses under identical conditions, with envelope-deficient (bald) pseudoviruses included as negative controls. Quantitative proteomic analysis identified significantly enriched proteins based on predefined enrichment and statistical criteria (Figure 2E). Across all three coronaviruses, 209 proteins were consistently enriched. After exclusion of proteins also enriched in bald-virus controls, 26 candidate Spike-associated host proteins were retained, including integrins, which have previously been implicated in coronavirus entry (Figure 2F–G) [24]. Functional annotation revealed that these candidates were mainly associated with membrane trafficking, protein synthesis, signal transduction, and cellular stress response pathways. We prioritized seven candidates for downstream validation based on their relatively higher abundance in the comparative proteomic analysis and their membrane association, given the specific aim of identifying cell-surface host factors involved in coronavirus Spike-mediated entry.

### PHB facilitates coronavirus Spike-mediated pseudovirus entry

**Figure 3.**
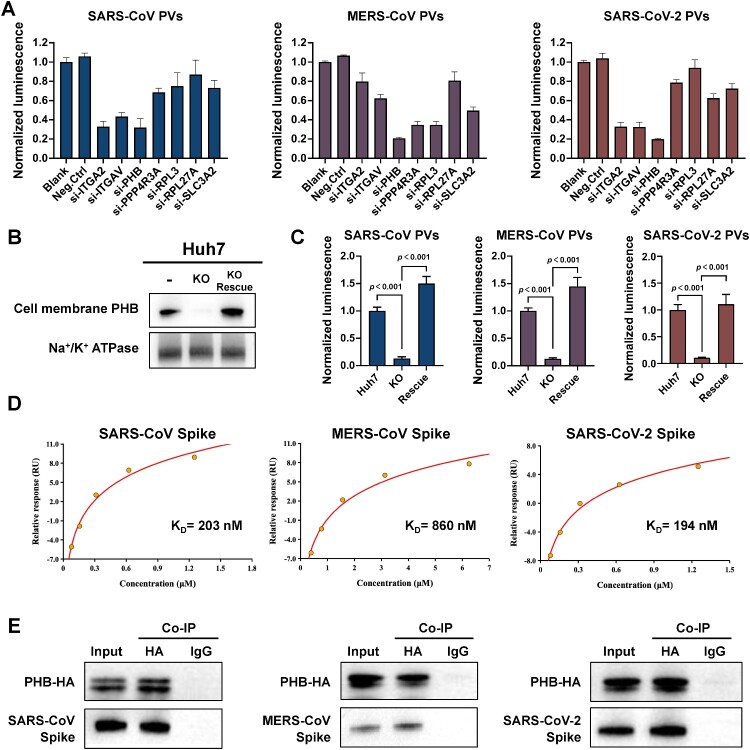
**PHB promotes coronavirus Spike-mediated pseudovirus entry and shows association with Spike proteins.** (A) siRNA screening of seven candidate host factors in Huh7 cells infected with SARS-CoV, MERS-CoV, or SARS-CoV-2 pseudoviruses. Infection efficiencies were quantified by normalized luminescence. PHB silencing produced the strongest inhibitory effect across all three pseudovirus systems. (B) Validation of PHB knockout (KO) and rescue cell models by immunoblotting of membrane fractions from parental, PHB-KO, and PHB-rescued Huh7 cells. Na^+^/K ^+^ -ATPase served as a membrane loading control. (C) Effects of PHB depletion and re-expression on coronavirus pseudovirus infection. Parental, PHB-KO, and PHB-rescued Huh7 cells were infected with SARS-CoV, MERS-CoV, or SARS-CoV-2 pseudoviruses, followed by quantification of infection by normalized luminescence. (D) Surface plasmon resonance analysis of interactions between recombinant PHB and coronavirus Spike proteins. Representative sensorgrams and fitted binding curves are shown together with the calculated apparent dissociation constants (K_D_). (E) Co-immunoprecipitation analysis of PHB association with coronavirus Spike proteins in cells. Lysates co-expressing PHB-HA and the indicated Spike proteins were immunoprecipitated with anti-HA antibody or IgG control, followed by immunoblot detection of PHB-HA and Spike proteins. Input lysates are shown as controls.

Among the seven membrane-associated candidates prioritized from the proteomic dataset, PHB was selected for further validation, as its knockdown (KD) most potently and consistently reduced pseudovirus infection across all three coronaviruses in Huh7 cells (Figure 3A). To systematically evaluate the role of PHB, we established stable PHB-knockout (KO) and rescue (KO + PHB) Huh7 cell lines, as well as transient siRNA-mediated knockdown and overexpression (OE) models (Figure 3B, Fig. S3A–C). In these models, experimental manipulation of membrane-associated PHB was accompanied by concordant changes in pseudovirus entry: PHB KO strongly impaired infection, which was restored upon PHB re-expression (Figure 3C). Similarly, siRNA knockdown reduced, whereas PHB overexpression enhanced, both cell-surface PHB levels and pseudovirus infectivity (Fig. S3D-F), indicating that the efficiency of Spike-mediated entry is positively associated with cell-surface PHB abundance. Consistent with these observations, anti-PHB antibodies inhibited SARS-CoV, MERS-CoV, and SARS-CoV-2 pseudovirus infection in a dose-dependent manner in Huh7 cells without detectable cytotoxicity (Fig. S4). Notably, this proviral effect was not restricted to a single coronavirus Spike, but was observed across SARS-CoV, MERS-CoV, and SARS-CoV-2, suggesting that PHB may represent a shared host factor for multiple human coronaviruses.

At the molecular level, SPR analysis demonstrated that recombinant PHB was capable of interacting with the Spike proteins of SARS-CoV, SARS-CoV-2, and MERS-CoV, with K_D_ values of 203, 194, and 860 nM, respectively (Figure 3D). Canonical receptor–Spike interactions were included as positive controls, while no detectable interaction was observed for PHB2 (Fig. S5). A PHB–Spike association was further supported by co-immunoprecipitation experiments performed in cells (Figure 3E).

### PHB promotes coronavirus Spike-mediated pseudovirus entry across epithelial cell systems

**Figure 4.**
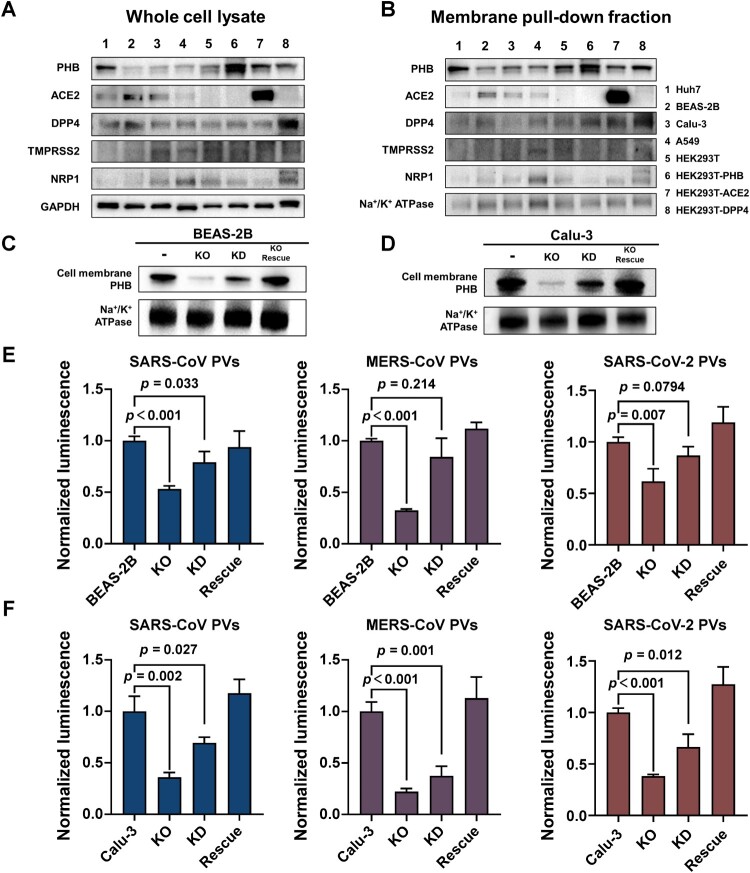
**PHB promotes coronavirus Spike-mediated pseudovirus entry in respiratory epithelial cell lines.** (A) Immunoblot analysis of PHB and selected coronavirus entry-related host factors in whole-cell lysates from the indicated cell lines. GAPDH was used as a loading control. (B) Immunoblot analysis of PHB and selected host factors in membrane pull-down fractions from the indicated cell lines. Na^+^/K ^+^ -ATPase served as a membrane fraction loading control. (C, D) Validation of PHB depletion and rescue in BEAS-2B (C) and Calu-3 (D) cells. Membrane fractions from parental cells, PHB-knockout (KO) cells, PHB-knockdown (KD) cells, and PHB-rescued cells were analyzed by immunoblotting for membrane-associated PHB. Na^+^/K ^+^ -ATPase was used as a membrane marker and loading control. (E, F) Effect of PHB depletion or restoration on pseudovirus infection in BEAS-2B (E) and Calu-3 (F) cells. Parental, PHB-KO, PHB-KD, and PHB-rescued cells were infected with pseudoviruses bearing the Spike proteins of SARS-CoV, MERS-CoV, or SARS-CoV-2, and infection efficiency was quantified by normalized luminescence. PHB loss reduced pseudovirus infection, whereas PHB re-expression restored susceptibility.

To determine whether the PHB-dependent entry phenotype observed in Huh7 cells could be reproduced in additional epithelial cell systems, we next examined BEAS-2B and Calu-3 cells. The proviral effect of PHB was reproducible in both BEAS-2B and Calu-3 cells, in which PHB was readily detected in both whole-cell lysates and membrane-associated fractions (Figure 4A, B). Consistent with the findings in Huh7 cells, depletion of membrane-associated PHB in both cell lines reduced infection by SARS-CoV, MERS-CoV, and SARS-CoV-2 pseudoviruses, whereas PHB re-expression restored susceptibility (Figure 4C–F). Notably, PHB knockout caused a stronger reduction in infection than partial knockdown, indicating a dosage-dependent contribution of PHB to Spike-mediated entry. Together, these findings demonstrate that the PHB-associated entry phenotype is reproducible across multiple epithelial cell systems.

### PHB-associated coronavirus entry is recapitulated in primary human airway epithelial cells and lung organoids

**Figure 5.**
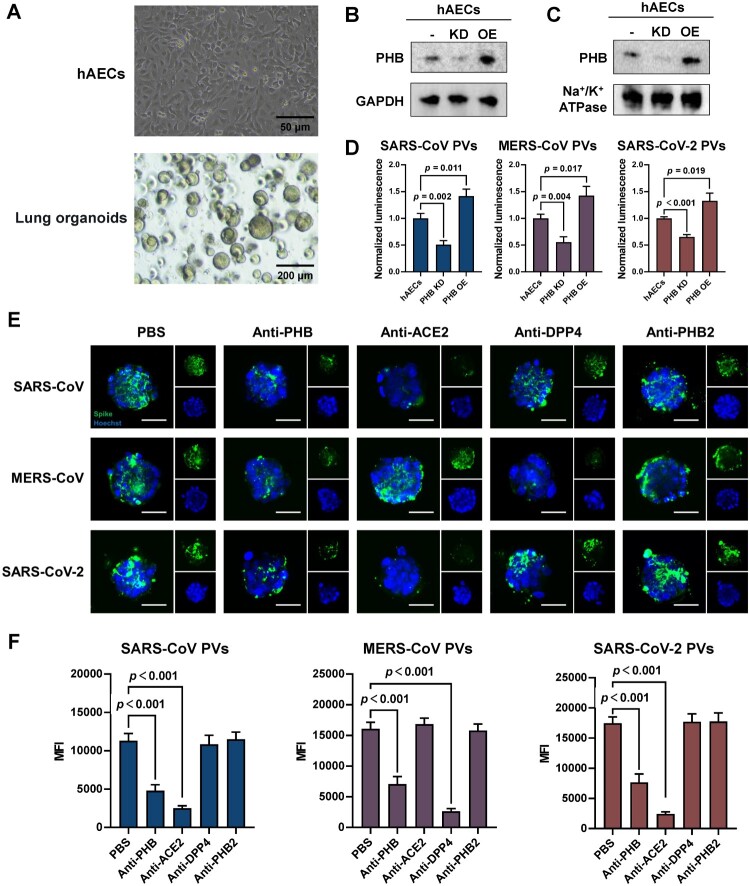
**PHB modulates coronavirus pseudovirus infection in primary human airway epithelial cells (hAECs) and human lung organoids.** (A) Representative images of primary hAECs and human lung organoids. (B, C) Immunoblot analysis of PHB expression in whole-cell lysates (B) and cell-surface fractions (C) from hAECs following PHB knockdown (KD) or overexpression (OE). GAPDH and Na^+^/K^+^-ATPase served as loading controls for whole-cell and membrane fractions, respectively. (D) Effects of PHB KD or OE on infection by SARS-CoV, MERS-CoV, and SARS-CoV-2 pseudoviruses in hAECs, quantified by normalized luminescence. PHB depletion reduced pseudovirus infection, whereas PHB overexpression restored susceptibility (n = 3). (E) Effects of antibody-mediated blockade of cell-surface PHB on coronavirus pseudovirus infection in human lung organoids. Antibodies against ACE2, DPP4, and PHB2 were included as controls. Pseudoviruses were labelled with AF488-NHS, and nuclei were counterstained with Hoechst. Scale bar, 50 μm. (F) Flow cytometric quantification of pseudovirus infection in dissociated lung organoid cells following antibody blockade. Infection levels were measured as mean fluorescence intensity (MFI) of AF488-positive signals (n = 3).

To further evaluate the physiological relevance of PHB-associated coronavirus entry beyond immortalized cell lines, we next established primary human airway epithelial cells (hAECs) and lung organoids derived from human lung tissues (Figure 5A). Public transcriptomic and protein atlas datasets revealed broad PHB expression across multiple human tissues, including respiratory-associated tissues (Fig. S6). Consistent with these findings, immunohistochemical analysis of normal human lung tissues from eight independent donors further demonstrated robust PHB expression in pulmonary epithelial structures, with detectable membrane-associated localization patterns (Fig. S7). Together, these observations support the physiological presence of PHB in human respiratory tissues.

We then examined whether membrane-associated PHB contributes functionally to coronavirus pseudovirus entry in primary human airway epithelial cells. Immunoblot analysis confirmed efficient PHB knockdown and overexpression in hAECs in both whole-cell lysates and membrane fractions (Figure 5B-C). Consistent with the observations in Huh7, BEAS-2B, and Calu-3 cells, modulation of PHB expression in hAECs produced concordant effects on pseudovirus susceptibility. PHB knockdown significantly reduced infection by SARS-CoV, MERS-CoV, and SARS-CoV-2 pseudoviruses, whereas PHB overexpression enhanced susceptibility across all three systems (Figure 5D). These findings indicate that the entry-promoting effect of PHB is reproducible in primary human respiratory epithelial cells and is not restricted to transformed or immortalized cell models.

To further assess the functional contribution of cell-surface PHB in a tissue-like respiratory model, we next performed antibody-blocking experiments in human lung organoids. AF488-labelled pseudoviruses readily associated with lung organoids under control conditions, whereas blockade with anti-PHB antibody markedly reduced pseudovirus binding across SARS-CoV, MERS-CoV, and SARS-CoV-2 systems (Figure 5E). As expected, anti-ACE2 preferentially inhibited SARS-CoV and SARS-CoV-2 pseudoviruses, whereas anti-DPP4 selectively suppressed MERS-CoV pseudovirus binding, supporting the specificity of the organoid infection model. In contrast, PHB2 blockade showed minimal effects (Figure 5E). Quantitative flow cytometric analysis of dissociated organoid cells further confirmed a significant reduction in pseudovirus-associated fluorescence intensity following PHB antibody treatment (Figure 5F). Notably, dose-dependent inhibition assays in hAECs demonstrated that anti-PHB antibodies suppressed pseudovirus infection with nanomolar-range potency without detectable cytotoxicity, comparable to the corresponding canonical receptor-blocking antibodies in their respective coronavirus systems (Fig. S8).

### A central region of the PHB extracellular domain is required for association with coronavirus Spike proteins

**Figure 6.**
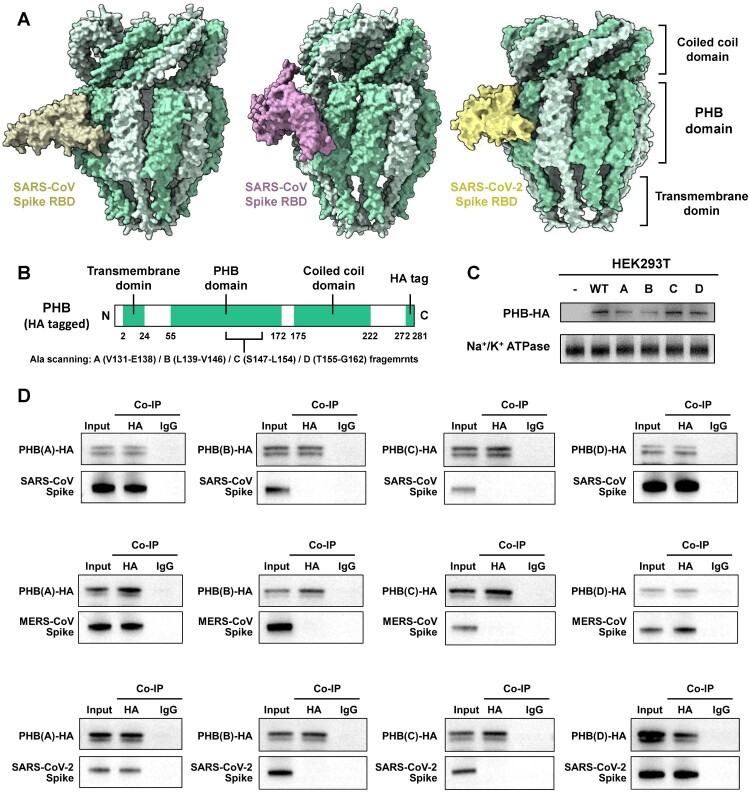
**Structural modelling and mutational analysis identify a central PHB region associated with coronavirus Spike interaction.** (A) AlphaFold 3-predicted models of PHB in complex with the RBDs of SARS-CoV, MERS-CoV, and SARS-CoV-2 Spike proteins. In all models, the RBD localized adjacent to the extracellular PHB domain rather than the transmembrane region, suggesting a shared interaction surface within the central extracellular region of PHB. (B) Domain organization of HA-tagged PHB and design of alanine-scanning mutants. PHB contains an N-terminal transmembrane domain, a central PHB domain, and a C-terminal coiled-coil domain. Four consecutive fragments spanning residues 131–162 were substituted by alanine mutagenesis: A (V131–E138), B (L139–V146), C (S147–L154), and D (T155–G162). (C) Immunoblot analysis of membrane fractions from HEK293 T cells expressing wild-type PHB-HA or the indicated alanine-scanning mutants. All mutants were detected in membrane-associated fractions. Na^+^/K ^+^ -ATPase served as a membrane loading control. (D) Co-immunoprecipitation analysis of associations between PHB mutants and coronavirus Spike proteins. HEK293 T cells co-expressing PHB-HA mutants and SARS-CoV, MERS-CoV, or SARS-CoV-2 Spike proteins were immunoprecipitated with anti-HA antibody or IgG control, followed by immunoblot detection of PHB-HA and Spike proteins. Mutations within fragments B and C markedly reduced Spike association across all three coronaviruses, whereas fragments A and D showed comparatively weaker effects.

To further define the PHB region involved in Spike association, we combined structural prediction with fragment-based mutagenesis. AF3 modelling predicted that the RBDs of SARS-CoV, MERS-CoV, and SARS-CoV-2 Spike proteins all localize adjacent to the central extracellular region of PHB, within or near the annotated PHB domain rather than the transmembrane or distal coiled-coil regions (Figure 6A). Based on this shared spatial distribution, alanine-scanning mutagenesis was performed across the PHB domain to identify residues potentially involved in Spike interaction. Alanine substitution across most regions of the PHB domain disrupted its plasma membrane localization, whereas substitutions within residues 131–162 had minimal effects on membrane enrichment. This region contains four consecutive alanine-scanning mutants (Figure 6B), all of which remained detectable in membrane-associated fractions (Figure 6C), indicating that subsequent differences in Spike interaction were unlikely to be attributable solely to altered membrane localization. Co-immunoprecipitation revealed a convergent effect across all three coronaviruses: mutations in fragments B (L139–V146) and C (S147–L154) consistently weakened the association of PHB with SARS-CoV, MERS-CoV, and SARS-CoV-2 Spike proteins, whereas mutations in fragments A (V131–E138) and D (T155–G162) had comparatively modest effects (Figure 6D). These data identify the central 139–154 aa region of PHB as a critical determinant for efficient association with multiple coronavirus Spike proteins and support the existence of a shared Spike-interacting hotspot within the extracellular PHB domain.

### PHB-associated coronavirus entry is linked to cholesterol-dependent membrane microdomains

**Figure 7.**
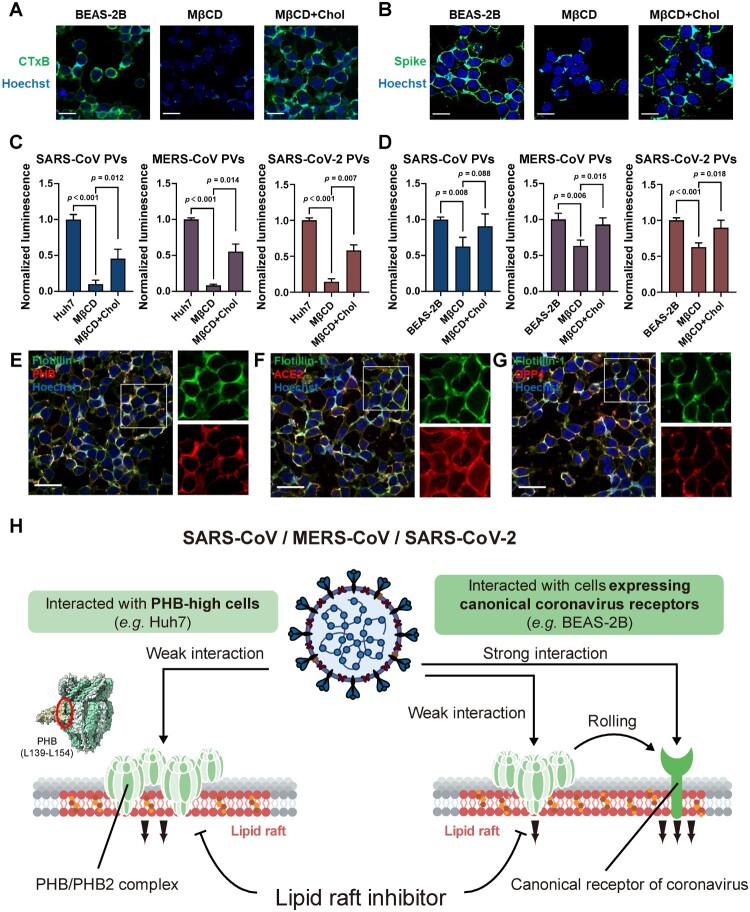
**PHB-associated coronavirus entry is sensitive to lipid raft disruption and linked to raft-associated membrane domains.** (A) Confocal imaging of the lipid raft marker cholera toxin subunit B (CTxB) in BEAS-2B cells under control conditions, following methyl-β-cyclodextrin (MβCD)-mediated cholesterol depletion, or after cholesterol replenishment (MβCD + Chol). Nuclei were stained with Hoechst. Scale bars, 20 μm. (B) Confocal analysis of coronavirus Spike binding in BEAS-2B cells under control, MβCD-treated, and cholesterol-replenished conditions. Nuclei were stained with Hoechst. Scale bars, 20 μm. (C, D) Effects of lipid raft disruption on SARS-CoV, MERS-CoV, and SARS-CoV-2 pseudovirus infection in Huh7 (C) and BEAS-2B (D) cells. Infection efficiencies were quantified by normalized luminescence following MβCD treatment with or without cholesterol replenishment. (E–G) Immunofluorescence analysis of the spatial relationship between the lipid raft marker Flotillin-1 and PHB (E), ACE2 (F), or DPP4 (G) in BEAS-2B cells. Insets show enlarged views of boxed regions. Scale bars, 50 μm. (H) Proposed model illustrating the contribution of PHB-associated membrane microdomains to coronavirus attachment and entry. Lipid raft disruption is proposed to impair both PHB-associated and canonical receptor-associated entry processes.

To explore the membrane context in which PHB facilitates coronavirus entry, we examined the role of lipid rafts. In BEAS-2B cells, treatment with methyl-β-cyclodextrin (MβCD) markedly reduced membrane staining of the lipid raft marker CTxB, whereas subsequent cholesterol replenishment restored this signal (Figure 7A), confirming effective disruption and partial reconstitution of cholesterol-dependent membrane microdomains. Under the same conditions, BEAS-2B cells were infected with SARS-CoV-2 pseudoviruses. The membrane-associated Spike signal was markedly reduced following MβCD treatment and was partially restored after cholesterol replenishment (Figure 7B), indicating that Spike binding is sensitive to lipid raft integrity. Consistent with this, MβCD significantly reduced infection by SARS-CoV, MERS-CoV, and SARS-CoV-2 pseudoviruses in both Huh7 and BEAS-2B cells, while cholesterol replenishment partially restored susceptibility in each case (Figure 7C-D). Thus, the entry-promoting effect observed in both PHB-high Huh7 cells and lung-related BEAS-2B cells depends, at least in part, on cholesterol-sensitive membrane microdomains. Immunofluorescence analyses in BEAS-2B cells further revealed clear spatial proximity between PHB and the lipid raft marker Flotillin-1 (Figure 7E), suggesting that the lipid raft dependence of viral infection may be mediated through direct association with PHB. Similar raft-associated localization patterns were also observed for ACE2 and DPP4 (Figure 7F-G), suggesting that PHB and canonical coronavirus receptors occupy related membrane microenvironments. Together, these data support a model in which PHB facilitates coronavirus attachment or entry within lipid raft-associated membrane domains and acts in a membrane environment related to canonical receptor-dependent entry pathways (Figure 7H).

## Discussion

In this study, we established a host-factor capture platform based on metabolic glycoengineering and photo-crosslinking to identify host factors involved in coronavirus entry. Using this strategy, we identified PHB as a conserved Spike-associated host factor shared by SARS-CoV, MERS-CoV, and SARS-CoV-2. Functional analyses across Huh7 cells, respiratory epithelial cell lines, primary human airway epithelial cells, and human lung organoids consistently demonstrated that reduction of membrane-associated PHB impaired pseudovirus infection, whereas PHB restoration or overexpression enhanced susceptibility. Together with the observed association between PHB and coronavirus Spike proteins, these findings support a role for PHB as a conserved membrane-proximal host factor linked to coronavirus entry.

PHB is a highly conserved scaffold protein localized to both the plasma membrane and mitochondrial inner membrane, where it participates in diverse cellular processes including proliferation, apoptosis, and signal transduction [25,26]. Previous studies have implicated PHB in the infection biology of multiple unrelated viruses, including Dengue virus [27], Chikungunya virus [28], hepatitis C virus [29], HIV [30], and EV71 [31], although the reported mechanisms vary substantially among viral systems. Collectively, these observations support the view that PHB functions less as a canonical viral receptor and more as a context-dependent membrane-associated host factor involved in regulating viral attachment or entry-associated processes [25–31]. In the context of coronaviruses, ACE2 and DPP4 remain the established primary receptors, whereas molecules such as heparan sulfate, NRP1, and AXL are generally considered attachment or entry-promoting cofactors [32–36]. Our findings place PHB closer to this latter category but suggest a distinct mechanistic role. Rather than functioning primarily in initial attachment, PHB appears to act within membrane-proximal entry environments, potentially contributing to the formation or stabilization of Spike-engaged entry microdomains. This interpretation is supported by several observations, including measurable associations between PHB and the Spike proteins of SARS-CoV, MERS-CoV, and SARS-CoV-2 in both SPR and co-immunoprecipitation assays, identification of residues 139–154 as a functionally important PHB region associated with Spike interaction, and the sensitivity of PHB-associated entry phenotypes to cholesterol depletion. Furthermore, spatial proximity analyses revealed close localization of PHB with lipid raft-associated membrane domains, supporting the possibility that PHB cooperates with canonical coronavirus receptors within organized membrane entry platforms. Collectively, these findings position PHB as a membrane-proximal auxiliary entry factor mechanistically distinct from previously described coronavirus cofactors.

The antibody-blocking experiments further suggest that PHB-associated entry processes may be therapeutically targetable. However, this possibility should be interpreted cautiously given the ubiquitous expression and pleiotropic cellular functions of PHB. In this regard, previously reported PHB-targeting flavagline derivatives displayed weak activity in our experimental system together with substantial cytotoxicity (Fig. S9) [37,38], indicating that they are unlikely to represent optimal inhibitors of PHB-associated coronavirus entry. Future therapeutic strategies may therefore benefit from selectively targeting extracellular PHB-associated interfaces or membrane-localized Spike-interacting regions while minimizing disruption of intracellular PHB functions. Such approaches may provide complementary host-directed antiviral strategies with potentially greater resilience to viral Spike evolution.

Several limitations of the present study should also be acknowledged. First, although the current work primarily relies on HIV-1–based pseudovirus systems rather than authentic live coronavirus infection models, the physiological relevance of the observed PHB-associated entry phenotype was strengthened through validation in primary hAECs, human lung organoids, and normal human lung tissues. Nevertheless, authentic coronavirus infection assays and in vivo studies will be necessary to further define the contribution of PHB during natural infection, replication, and pathogenesis. Second, although residues 139–154 were identified as a critical extracellular PHB region associated with Spike interaction, the precise molecular interface remains unresolved and will require higher-resolution structural and mutational analyses. Third, the functional interplay among PHB, canonical coronavirus receptors, and cholesterol-rich membrane microdomains remains incompletely understood. Despite these limitations, our findings expand current understanding of coronavirus host factor utilization by identifying PHB as a conserved Spike-associated host factor whose functional relevance is reproducible across transformed cell lines, primary respiratory epithelial cells, and lung organoid systems.

## Supplementary Material

Supplementary information.docx
