# Supplementary material for: Identification of Prohibitin (PHB) as an Entry Factor of Highly Pathogenic Coronaviruses Using Metabolic Glycoengineering and Photo-Crosslinking Techniques
Source: Emerg Microbes Infect. 2026 Jun 8;15(1):2686461. doi: 10.1080/22221751.2026.2686461 (PMC13288833; doi:10.1080/22221751.2026.2686461)
Supplement: Supplementary information.docx [file TEMI_A_2686461_SM8317.docx]

Supplementary information

**Identification of Prohibitin (PHB) as an Entry Factor of Highly Pathogenic Coronaviruses Using Metabolic Glycoengineering and Photo-Crosslinking Techniques**

Xingxing Zhu^a,b,1^, Xiaoyang Wang ^a,1^, Yiming Wang^a,1^, Xinchen Wang^a^, Yuanhao Li^a^, Liwen Hua^a^, Honggao Duan^a^, Rongding Yan^a^, Tao Yuan^a^, Jing Li^f^, Yeshuang Yuan^g^, Bo Zhang^f^, Demin Zhou^a,c,d,e*^, Sulong Xiao ^a,c,d,e*^

**Contents**

**Fig S1.** Optimization of SARS-CoV-2 pseudovirus infection time in HEK293T-ACE2 cells. **S1**

**Fig S2.** Western blot of SARS-CoV-2 pseudoviruses subjected to photo-crosslinking. **S2**

**Fig S3.** PHB enhances infection of multiple coronavirus pseudoviruses in Huh7 cells. **S3**

**Fig S4.** Dose-dependent inhibition of coronavirus pseudovirus infection in Huh7 cells. **S5**

**Fig S5.** Binding analysis of coronavirus Spike proteins to candidate host receptors by SPR. **S6**

**Fig S6.** Transcriptomic and protein expression landscape of PHB across human tissues. **S7**

**Fig S7.** Immunohistochemical analysis of PHB expression in normal human lung tissues from eight independent donors. **S8**

**Fig S8.** Dose-dependent inhibition of coronavirus pseudovirus infection in hAECs. **S10**

**Fig S9.** SPR analysis of Rocaglamide and Silvestrol binding to PHB protein, along with their cytotoxicity in Huh7 cells. **S11**

**Fig S10.** Unedited full-length western blot images in this study. **S12**

**
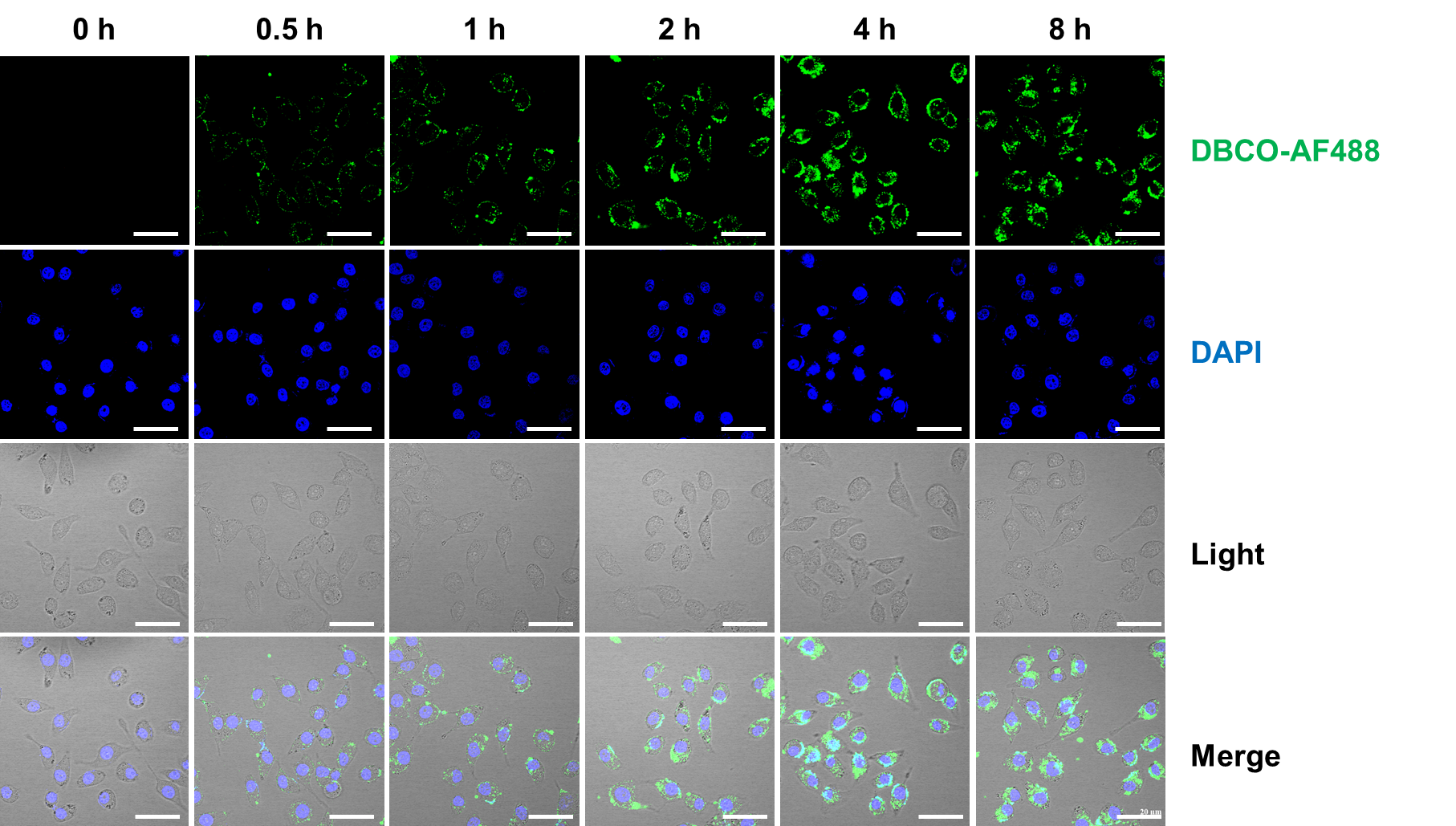
**

**Fig S1. Optimization of SARS-CoV-2 pseudovirus infection time in HEK293T-ACE2 cells.**

Pseudoviruses were labeled with DBCO-AF488 (green), and nuclei were stained with DAPI (blue). Scale bar, 50 μm.

**
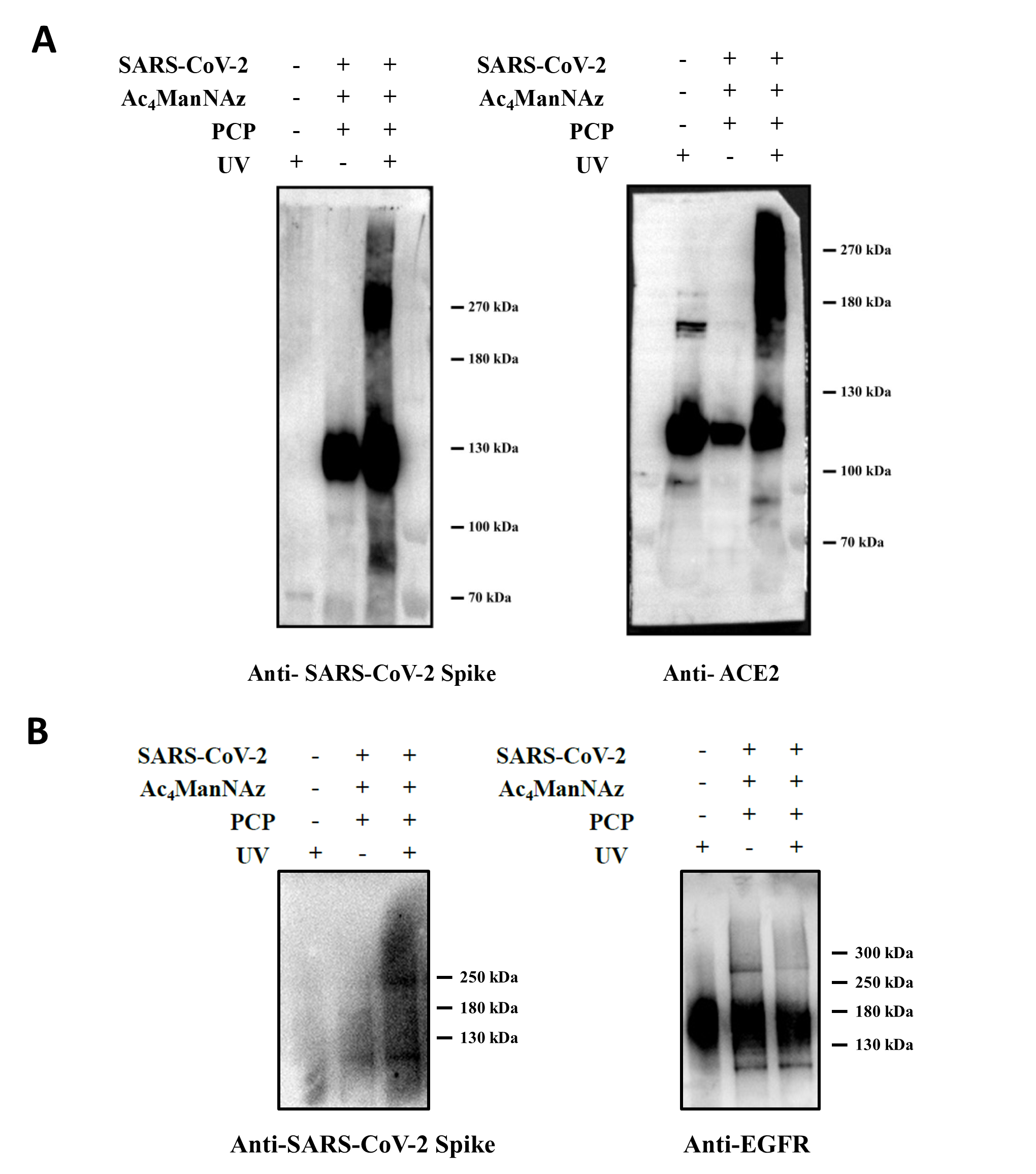
**

**Fig S2. Western blot of SARS-CoV-2 pseudoviruses subjected to photo-crosslinking.**

(A) Pseudoviruses (MOI = 10) were incubated with HEK293T-ACE2 cells at 4°C for 2 h to inhibit viral internalization and enrich particles on the cell surface. In the UV-irradiated group, diazirine moieties in the trifunctional probe were activated to form covalent crosslinks between Spike proteins and adjacent host membrane proteins, capturing Spike–host complexes. The non-irradiated group was served as a control.

(B) EGFR did not show an upward band shift before or after photo-crosslinking irradiation.

**
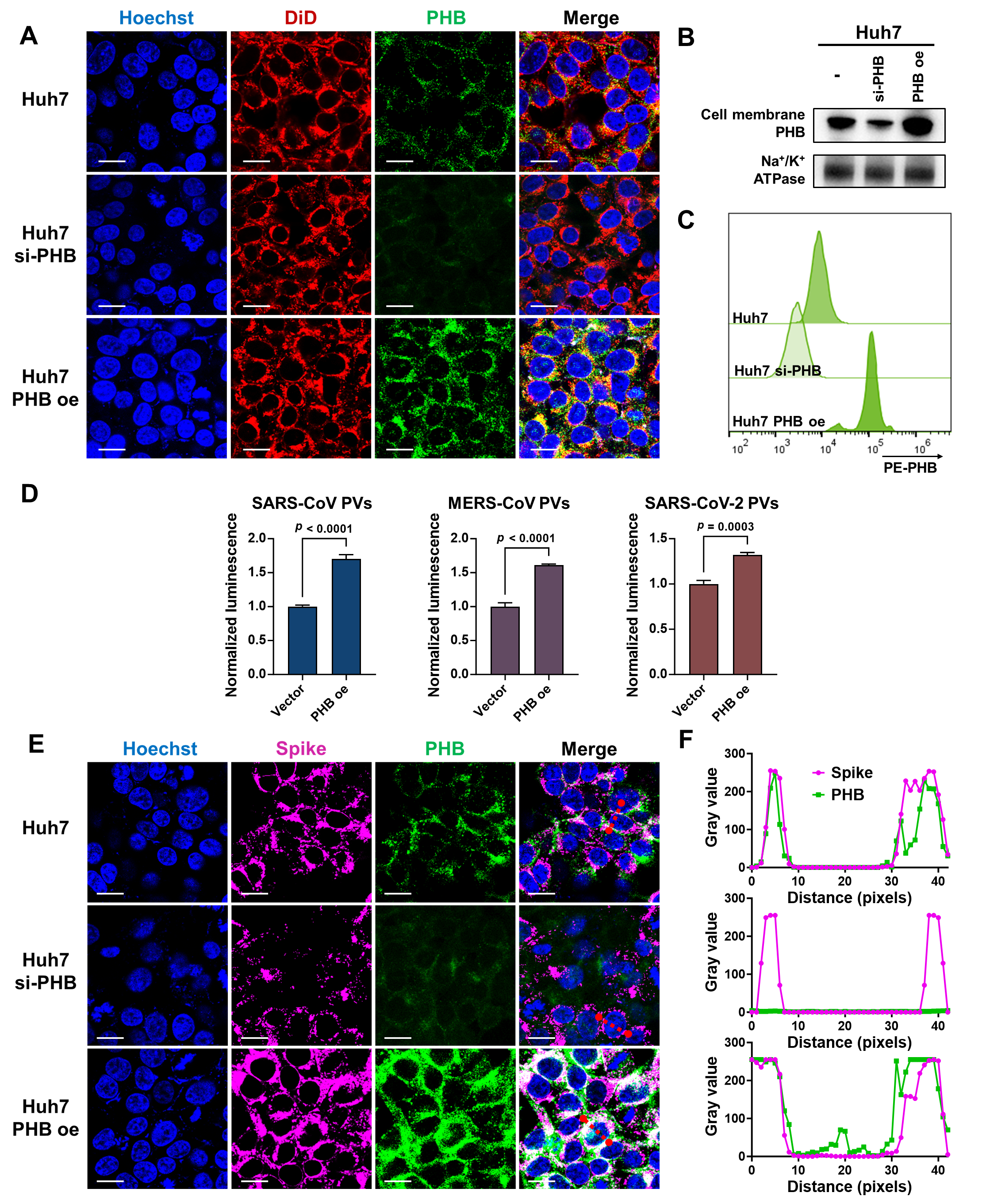
**

**Fig S3. PHB enhances infection of multiple coronavirus pseudoviruses in Huh7 cells.**

(A) Immunofluorescence analysis of membrane-associated PHB expression in normal Huh7 cells, PHB knockdown cells, and PHB-overexpressing cells. Nuclei were stained with Hoechst (blue), cell membranes with DiD (red), and PHB with anti-PHB antibodies (green). Scale bar, 20 μm.

(B–C) Analysis of membrane-associated PHB expression in normal Huh7 cells, PHB knockdown cells, and PHB-overexpressing cells by western blotting (B) and flow cytometry (C).

(D) Effects of PHB overexpression on infectivity of SARS-CoV (navy), MERS-CoV (purple), and SARS-CoV-2 (brown) pseudoviruses in Huh7 cells. Data are presented as mean ± SD (n = 3). Student’s t-tests were used for group comparisons.

(E) Immunofluorescence analysis of colocalization between SARS-CoV-2 Spike proteins and PHB on the surface of Huh7 cells. Spike proteins were stained with anti-Spike antibodies (magenta), PHB with anti-PHB antibodies (green), and nuclei with Hoechst (blue). Scale bar, 20 μm.

(F) Quantitative colocalization analysis corresponding to panel (E). Regions of interest are indicated by red dashed outlines in the merged images.


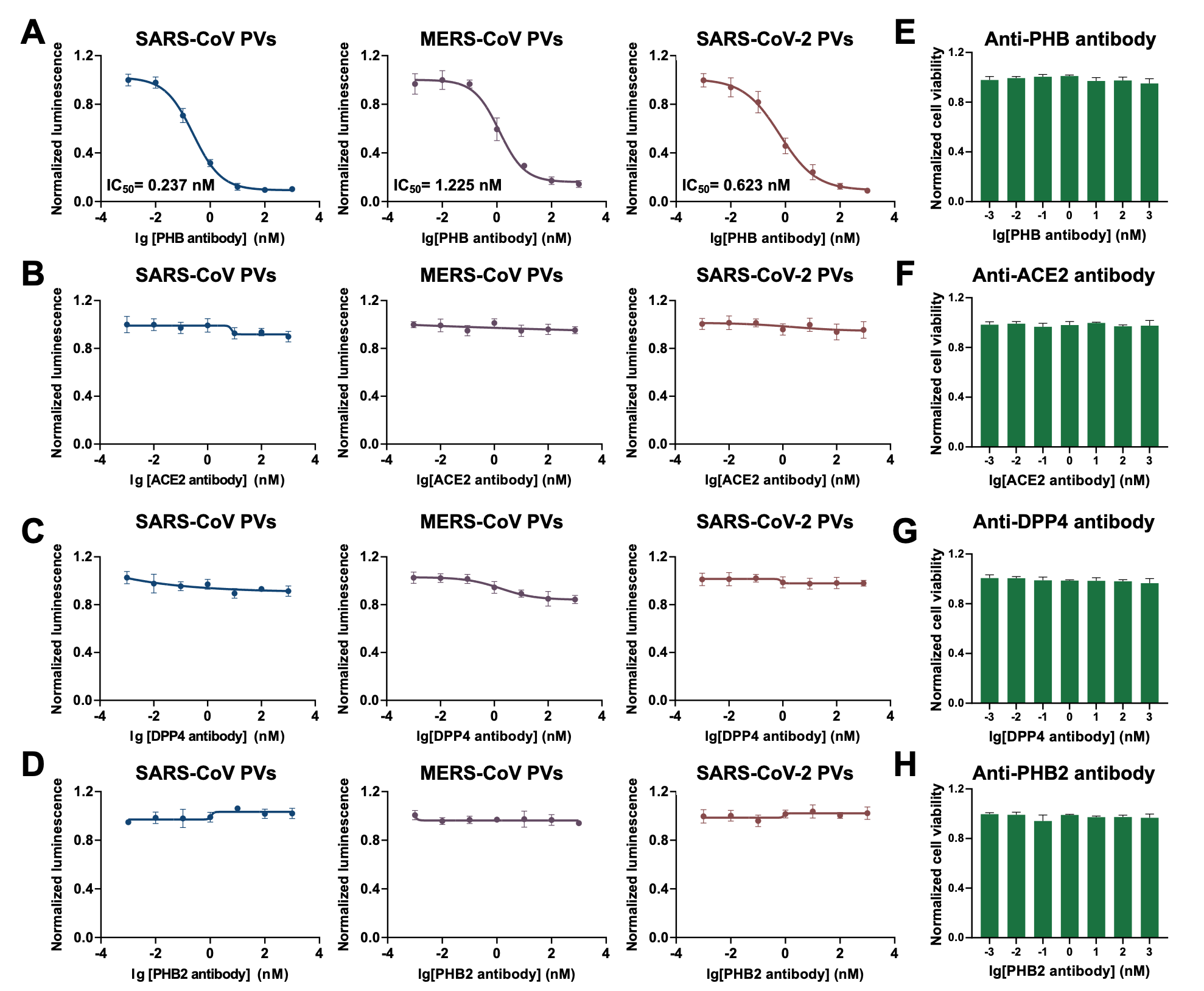


**Fig S4. Dose-dependent inhibition of coronavirus pseudovirus infection in Huh7 cells.**

(A–D) Dose-dependent inhibition of coronavirus pseudovirus infection by antibodies against PHB (A), ACE2 (B), DPP4 (C), and PHB2 (D). (E–H) Corresponding cell viability analyses for anti-PHB (E), anti-ACE2 (F), anti-DPP4 (G), and anti-PHB2 (H) antibodies.

**
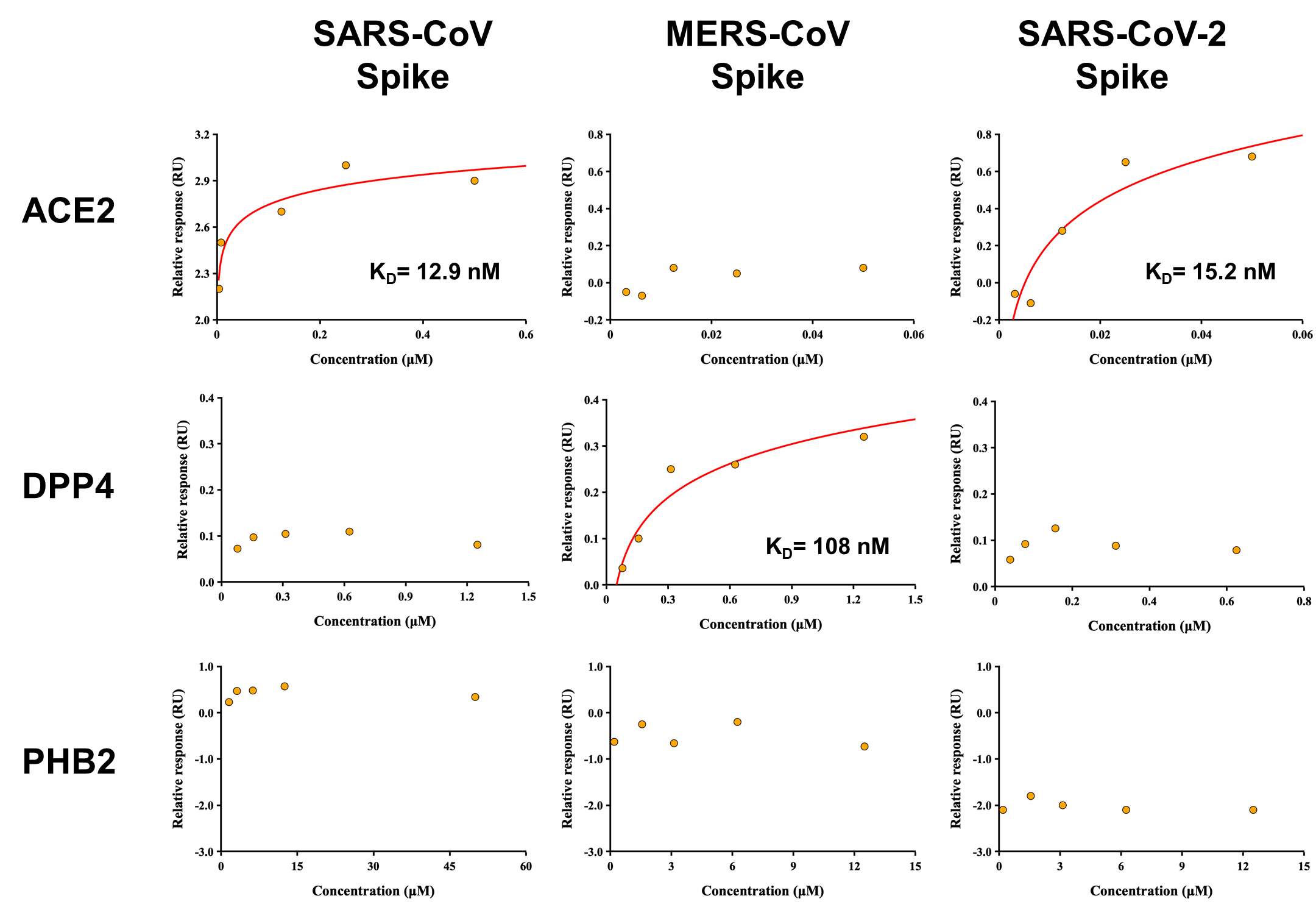
**

**Fig S5.** **Binding analysis of coronavirus Spike proteins to candidate host receptors by SPR.**

Surface plasmon resonance (SPR) was used to evaluate the interactions of SARS-CoV, MERS-CoV, and SARS-CoV-2 Spike proteins with ACE2, DPP4, and PHB2. Sensorgrams were fitted using a steady-state binding model to estimate equilibrium dissociation constants (K_D_ where applicable. Detectable binding was observed between SARS-CoV Spike and ACE2 (K_D_= 12.9 nM), SARS-CoV-2 Spike and ACE2 (K_D_ = 15.2 nM), and MERS-CoV Spike and DPP4 (K_D_ = 108 nM), consistent with their established receptor usage. In contrast, no appreciable binding signal was detected between PHB2 and any of the three Spike proteins, nor between non-cognate receptor–Spike pairs under the tested conditions.

**
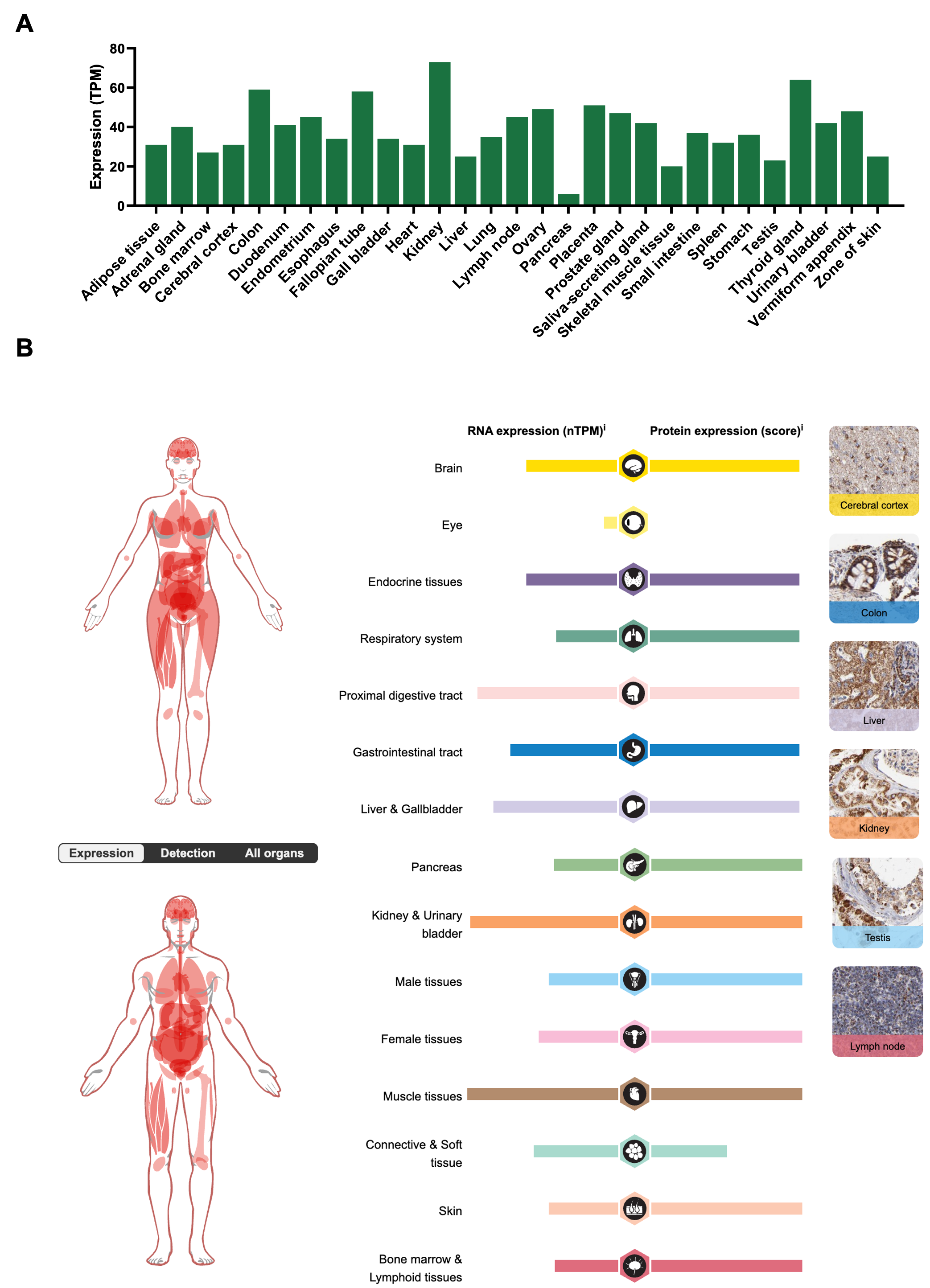
**

## **Fig S6. Transcriptomic and protein expression landscape of PHB across human tissues.**

(A) PHB transcriptomic expression across human tissues based on the Expression Atlas database, shown as transcripts per million (TPM).

(B) PHB expression landscape across human tissues and organs based on the Human Protein Atlas database. The body map illustrates the overall distribution of PHB expression in female and male tissues. The corresponding panel summarizes RNA expression levels, protein expression patterns, and representative immunohistochemical staining across major organ systems, demonstrating broad PHB expression in multiple human tissues, including respiratory-associated tissues.

**
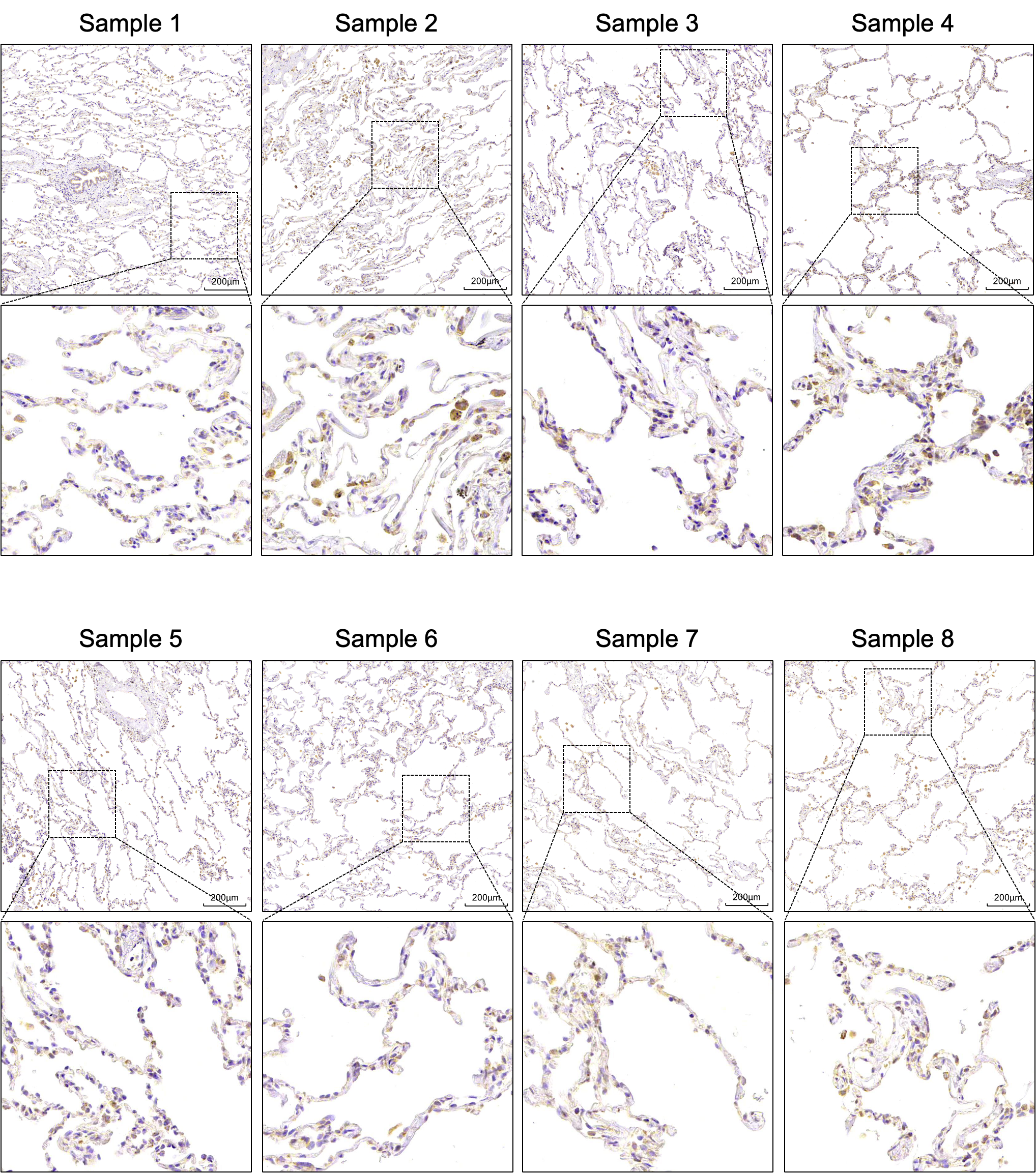
**

**Fig S7. Immunohistochemical analysis of PHB expression in normal human lung tissues from eight independent donors.**

PHB expression was detected in both the cytoplasm and cell membrane of pulmonary cells.

**
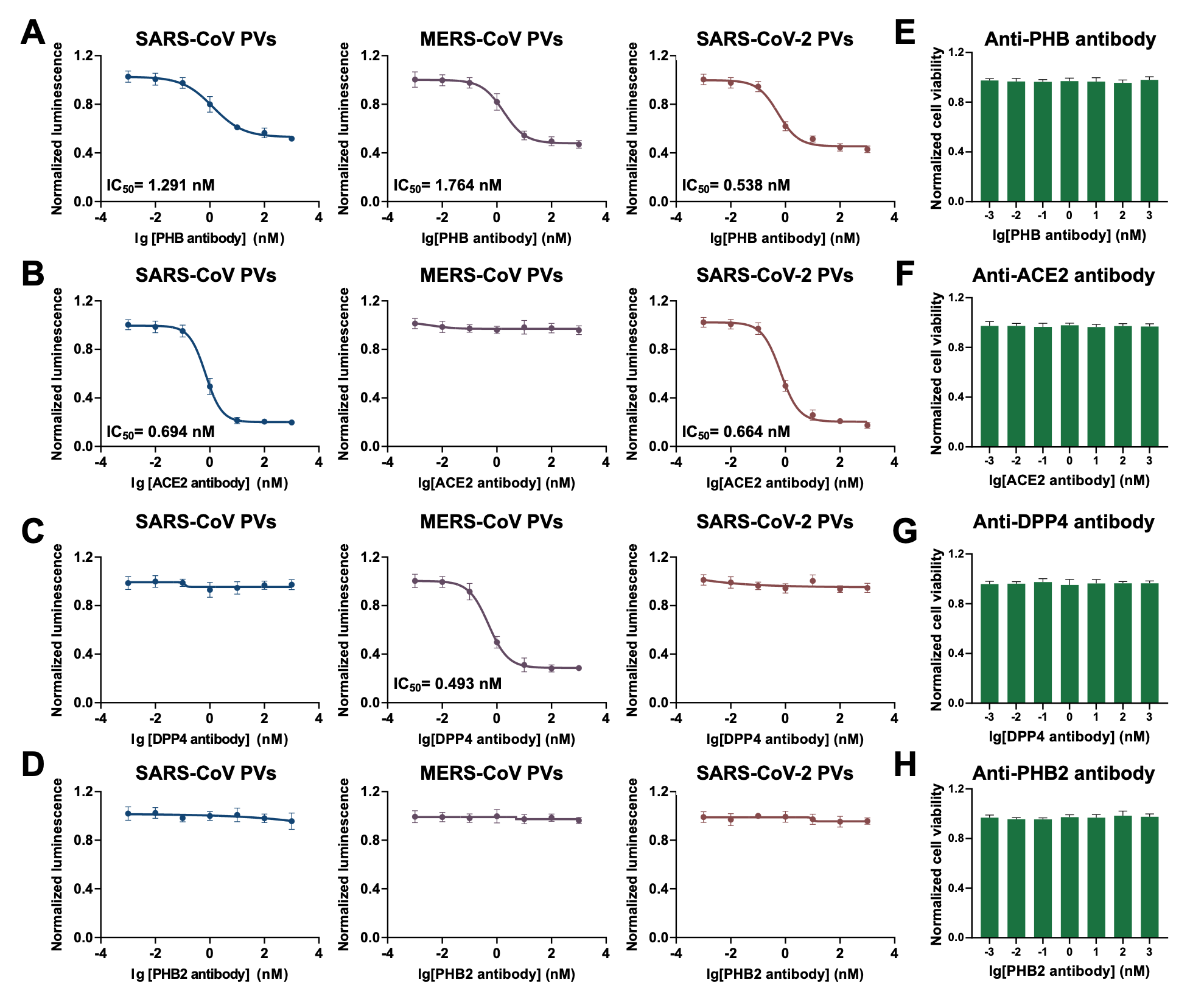
**

**Fig S8. Dose-dependent inhibition of coronavirus pseudovirus infection in hAECs.**

(A–D) Dose-dependent inhibition of coronavirus pseudovirus infection by antibodies against PHB (A), ACE2 (B), DPP4 (C), and PHB2 (D). (E–H) Corresponding cell viability analyses for anti-PHB (E), anti-ACE2 (F), anti-DPP4 (G), and anti-PHB2 (H) antibodies.

**
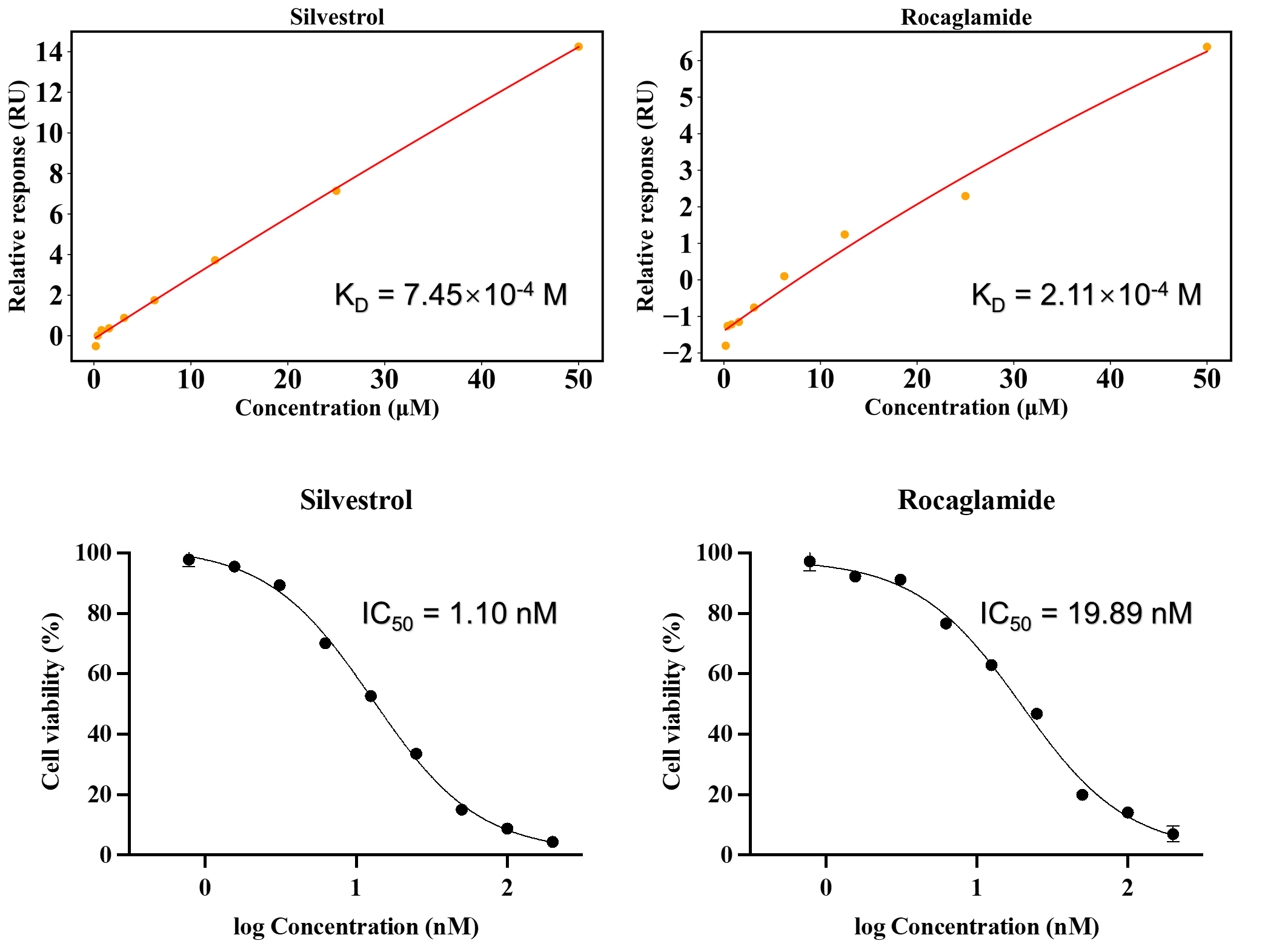
**

**Fig S9. SPR analysis of Rocaglamide and Silvestrol binding to PHB protein, along with their cytotoxicity in Huh7 cells.**

Half-maximal inhibitory concentration (IC_50_) values were determined using GraphPad Prism Software 9.0.

**
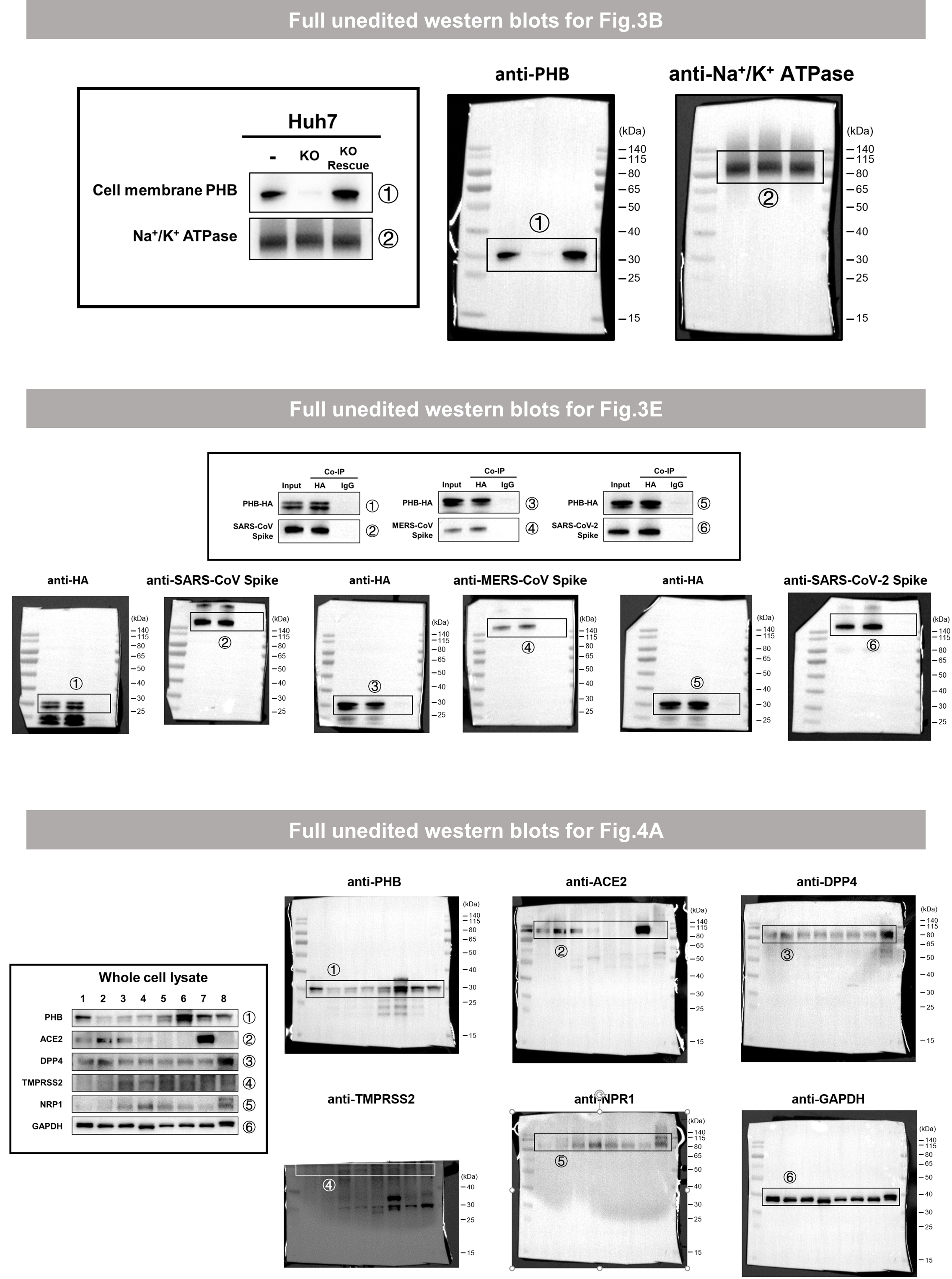
**

**(Continued)**

**
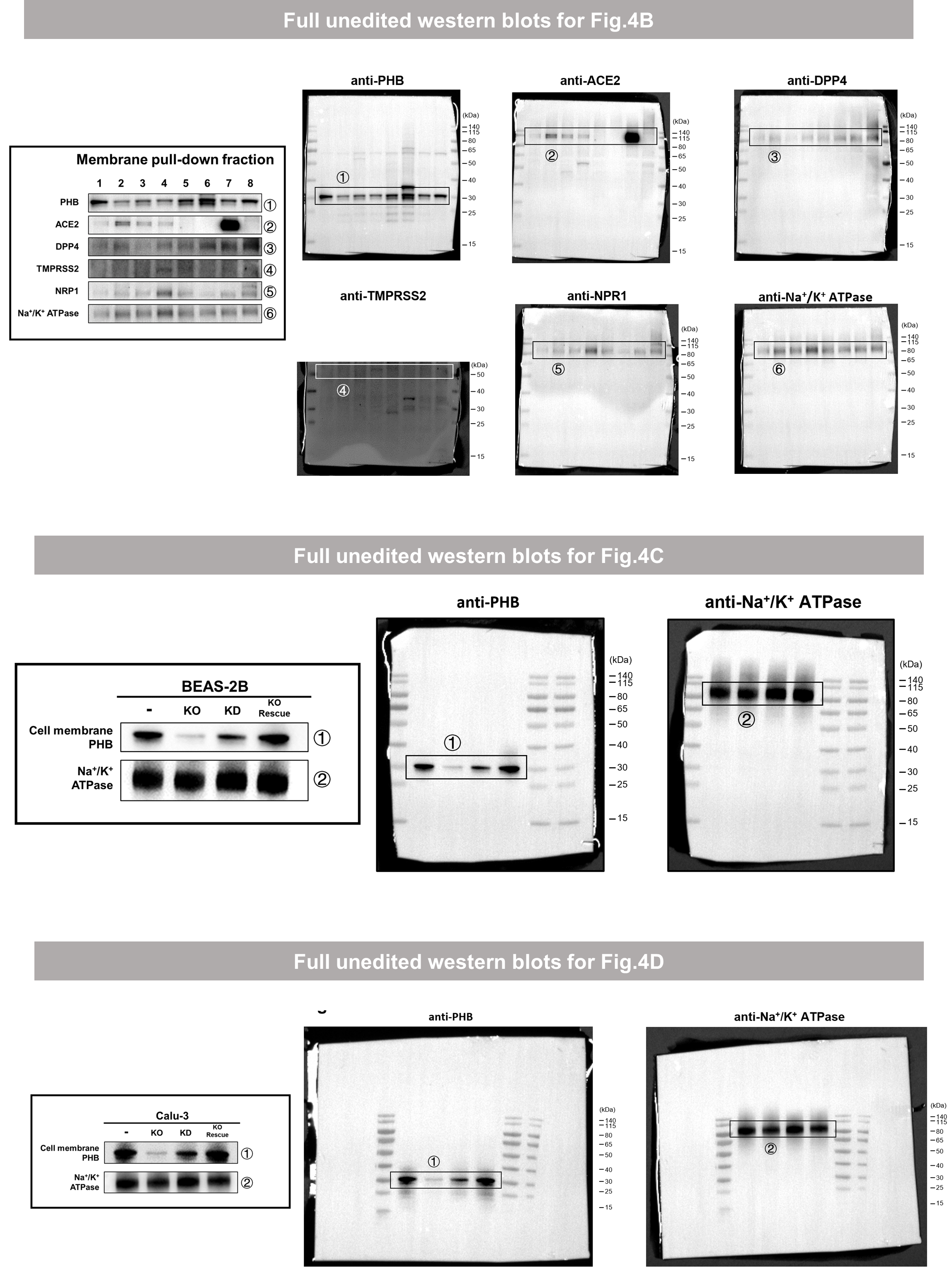
**

**
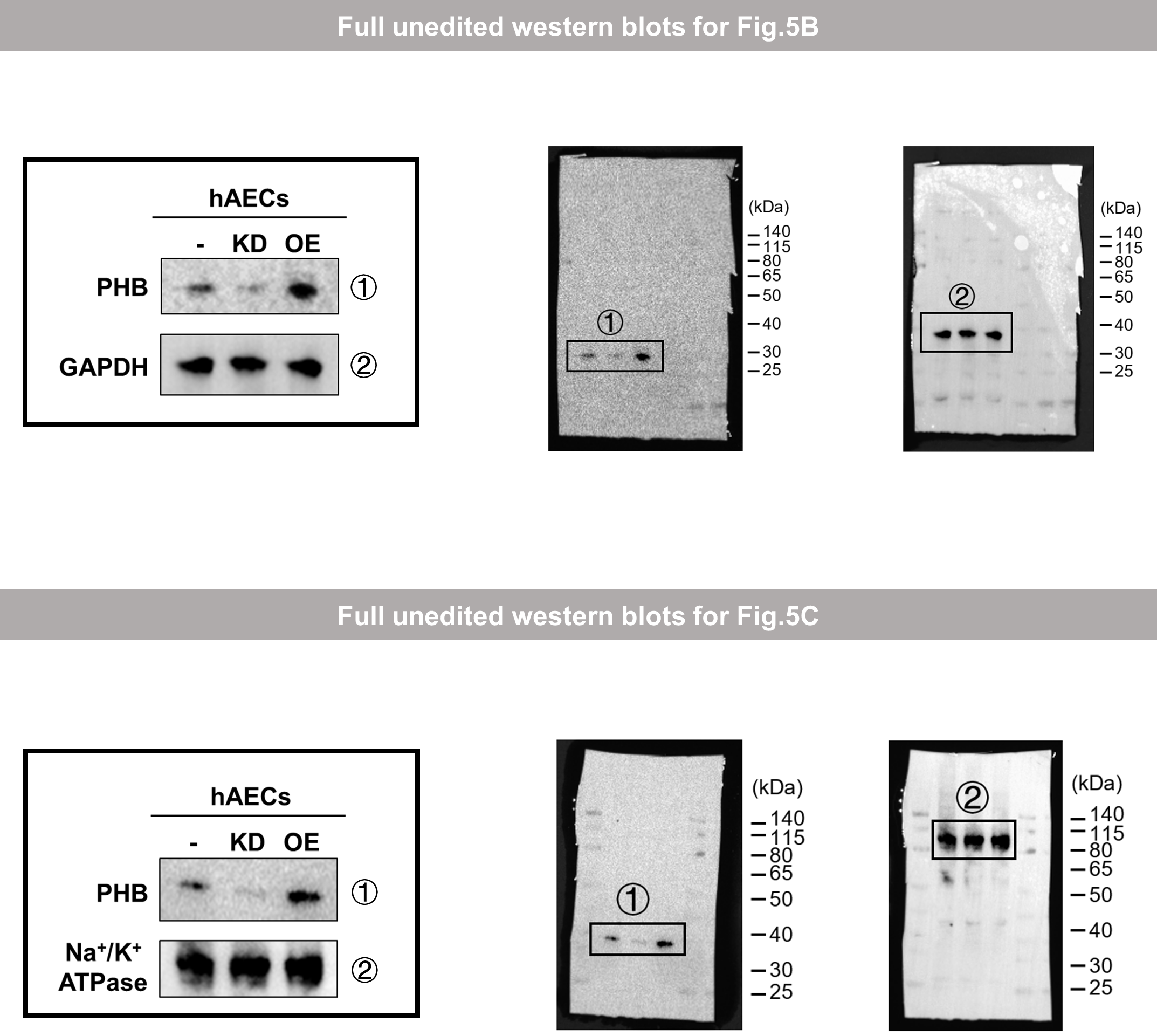
(Continued)**

**(Continued)**

**
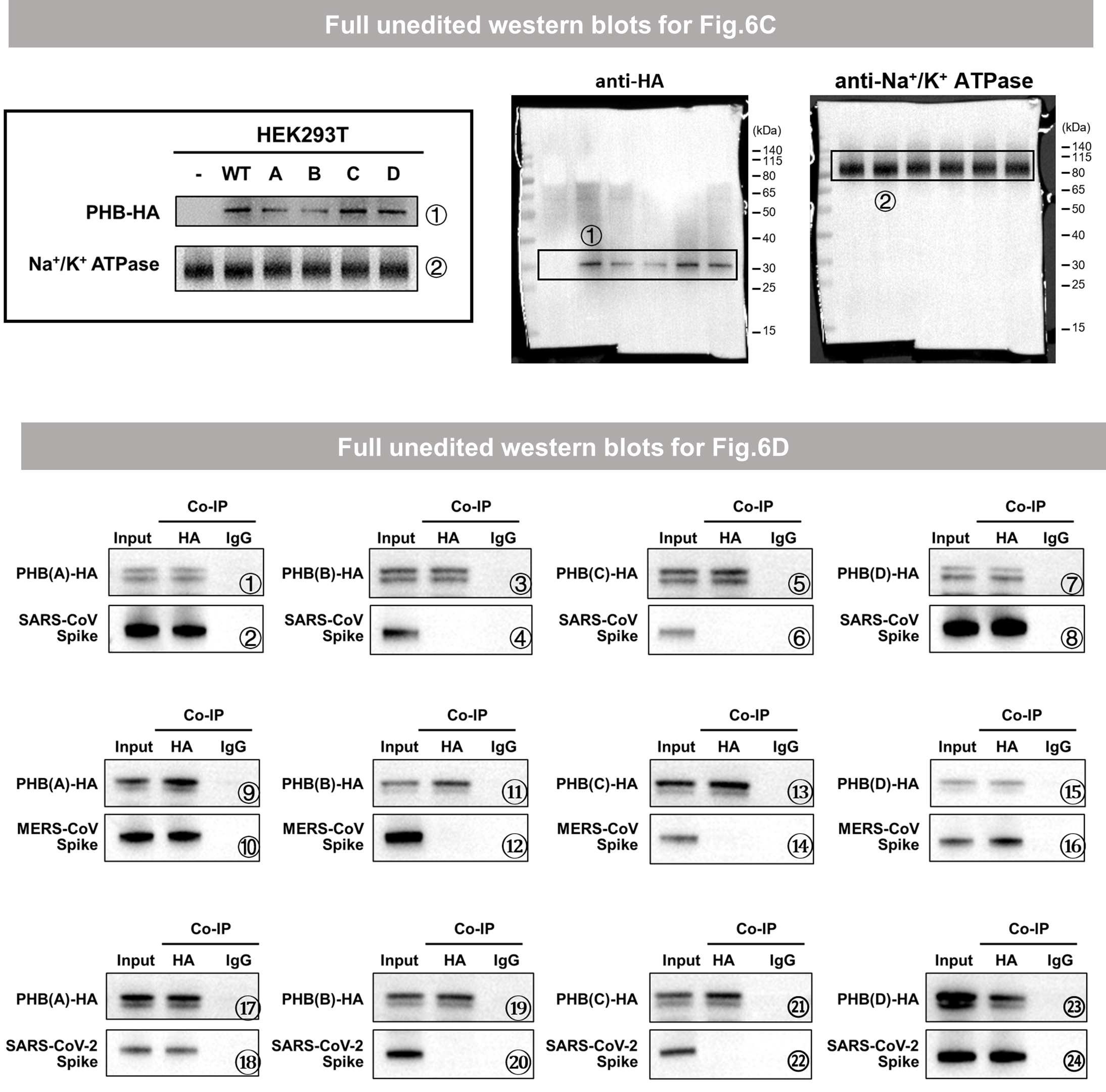
**

**
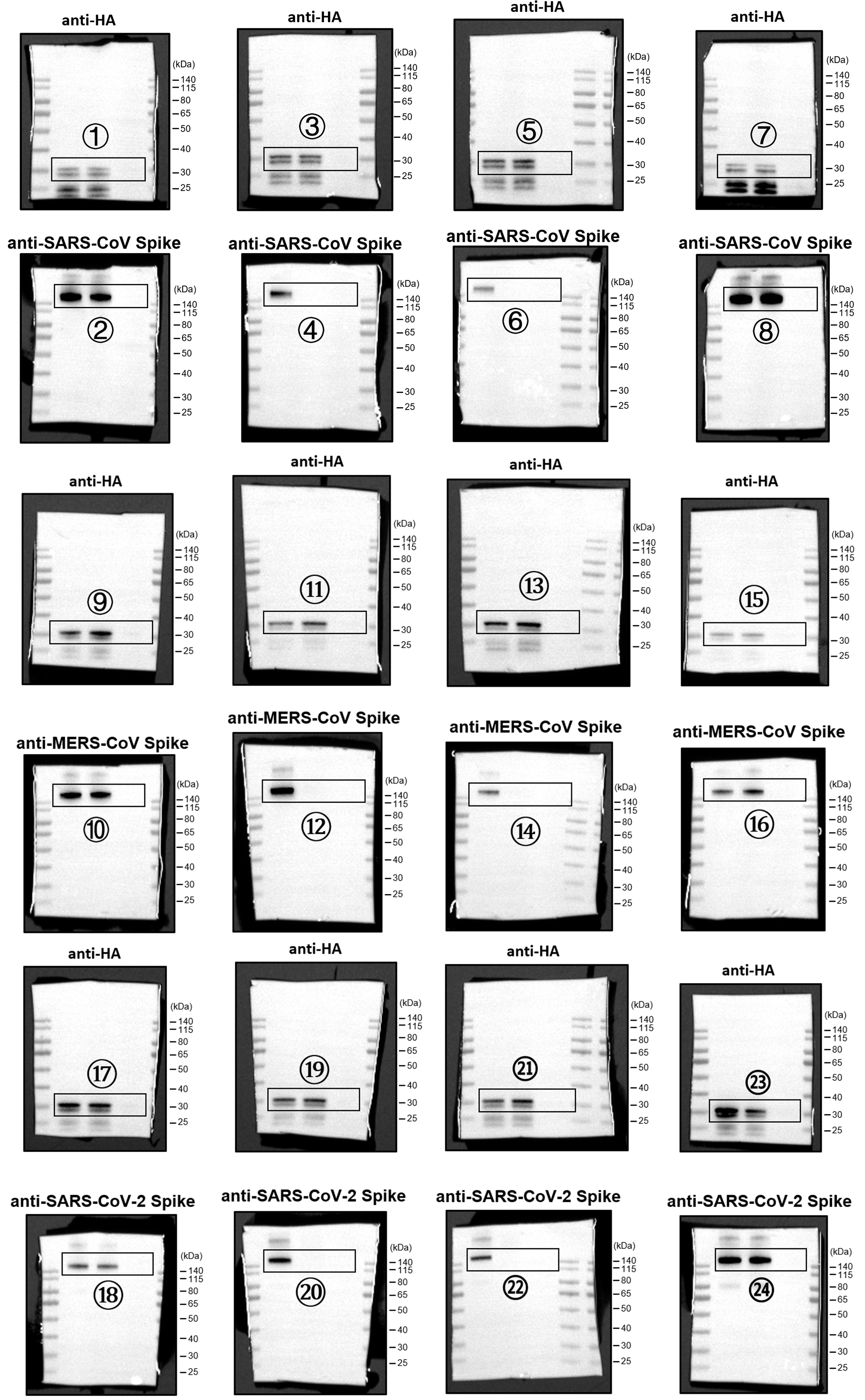
(Continued)**

**
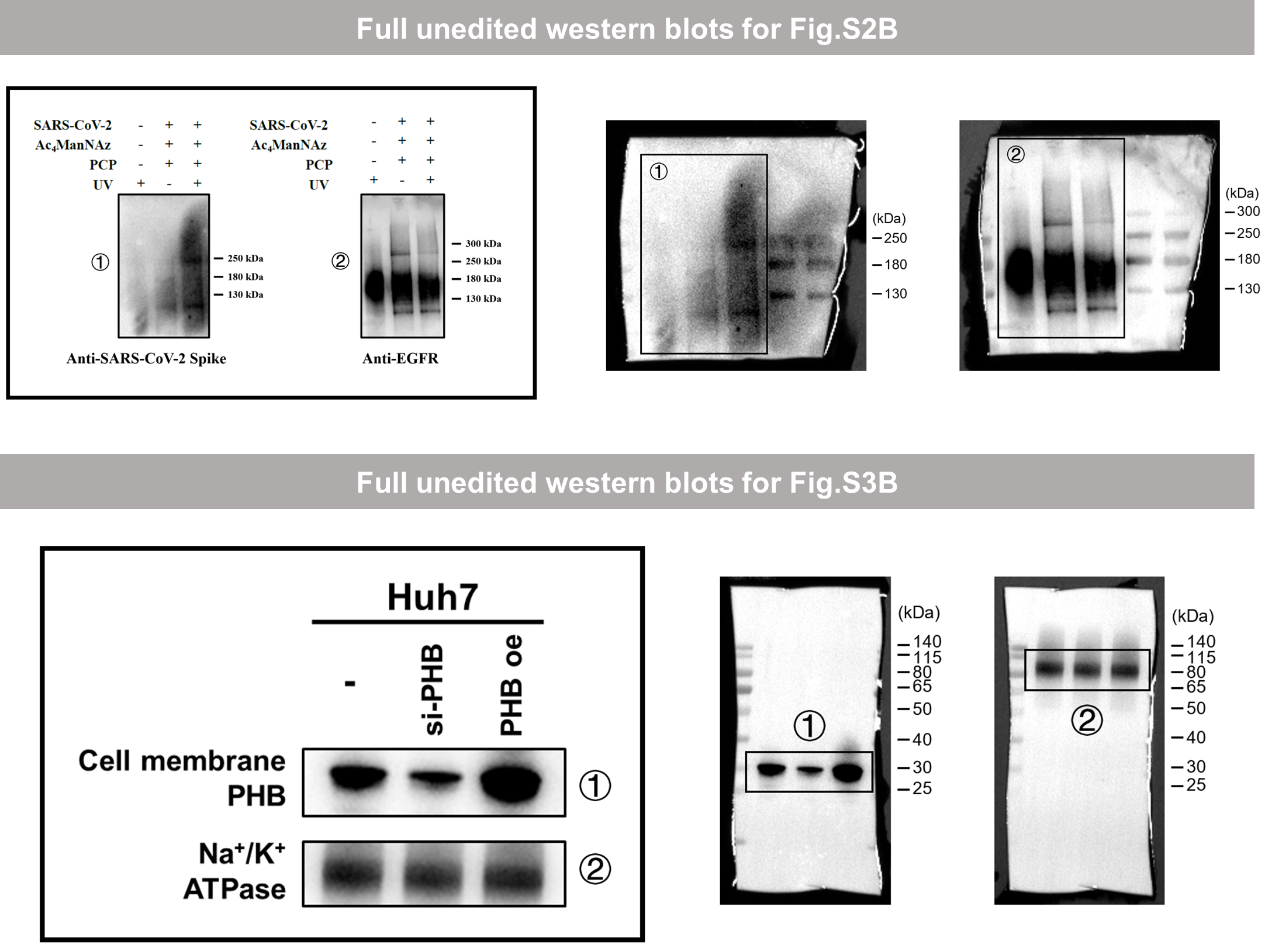
(Continued)**

**Fig S10. Unedited full-length western blot images for all experiments in this study.**
